# Coarse-Grained Nonadiabatic Dynamics in Disordered Condensed Phases

**DOI:** 10.1021/acs.jctc.6c01363

**Published:** 2026-09-04

**Authors:** Zailing Song, Zengkui Liu, Fei Xia, Yuwei Zhang, Xiang Sun

**Affiliations:** † Division of Arts and Sciences, 447103 NYU Shanghai, 567 West Yangsi Road, Shanghai 200124, China; ‡ Department of Chemistry, New York University, New York, New York 10003, United States; § 12655 NYU-ECNU Center for Computational Chemistry at NYU Shanghai, 3663 Zhongshan Road North, Shanghai 200062, China; ∥ School of Chemistry and Molecular Engineering, 12534 East China Normal University, Shanghai 200062, China; ⊥ School of Chemistry and Materials Science, Nanjing Normal University, Nanjing 210000, China

## Abstract

The mechanisms underlying critical energy-gap fluctuations and nonadiabatic dynamics in disordered condensed phases remain elusive, and their accurate simulation poses a challenge due to the inherent complexity of these systems. In this study, we make the first attempt to leverage highly efficient coarse-grained (CG) models to investigate these fundamental mechanisms. Employing a rigorous bottom-up parameterization approach, we construct CG models for two prototypical systems: a carotenoid–porphyrin–fullerene triad in tetrahydrofuran and a perylene diimide dimer in acetonitrile. Using nonadiabatic semiclassical mapping dynamics, we evaluate a hierarchy of representations spanning fully atomistic explicit-molecule simulations, intermediate CG resolutions, and highly reduced multistate harmonic (MSH) models to assess their impact on energy-gap fluctuations and nonadiabatic properties. A distinct advantage of these explicit-molecule simulations is their ability to yield time-dependent radial distribution functions, revealing the detailed molecular picture of localized solvent rearrangement upon photoinduced charge transfer. Our results confirm that with appropriate coarse-graining of the solvent and solute, the resulting CG models faithfully preserve the structural responses, pairwise reorganization energies, and electronic population dynamics of the atomistic benchmark. Conversely, aggressive 2-heavy-atom solute mapping severely distorts local nonbonded environments. Importantly, MSH models parameterized from structurally valid CG simulations successfully reproduce explicit-molecule dynamics while discarding tens of thousands of degrees of freedom. This work provides a new, highly efficient theoretical foundation for scaling nonadiabatic dynamics simulations to larger and more complex condensed-phase systems.

## Graphical abstract

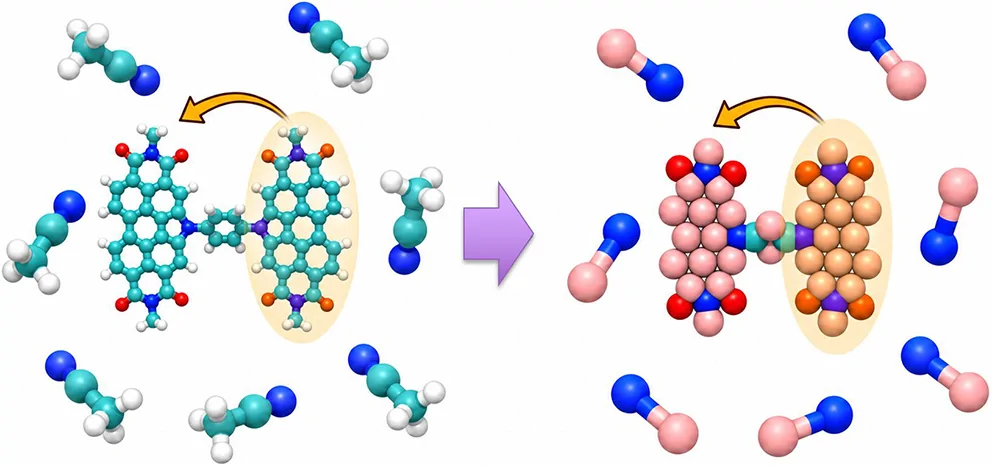

## 1 Introduction

Photoinduced charge transfer (CT) is a core process in solar energy conversion, playing a particularly critical role in organic photovoltaic (OPV) materials and artificial photosynthesis.^1–6^ A typical CT sequence initiates with light absorption: light-harvesting molecules are excited from their ground state to a locally excited state. The resulting excitons diffuse to the donor/acceptor interface, where CT occurs and free charge carriers are formed.^7–10^ These carriers may eventually be transported to the electrodes or recombine.^11–14^ To rationally design more efficient OPV materials, a rigorous molecular understanding of the nonadiabatic dynamics underlying these processes in disordered condensed phases, such as bulk-heterojunction blends, is absolutely necessary.

The condensed phase introduces significant complexities to this problem. A CT complex is not surrounded by a static background; it is embedded in a fluctuating solvent or molecular environment that actively participates in the dynamics. The surrounding molecules strongly affect the nonadiabatic mechanism, and intermolecular interactions dictate the transition pathways. Disorder in these systems manifests in two distinct forms. Static disorder arises from variations in local packing morphology or molecular topology, which creates different local environments.^2,15–21^ Dynamic disorder is driven by the thermal motions of the molecules and electronic-vibrational coupling.^22–26^ Because of this dual nature of disorder, photoinduced CT in realistic condensed-phase systems cannot be described by the electronic structure of the isolated molecule alone. The environmental motion must be explicitly included as part of the dynamics.

In recent years, several dynamical methods have been developed to treat these large-scale electronic transitions. Ehrenfest mean-field (MF) dynamics^27^ and fewest-switches surface hopping^28^ are two widely used mixed quantum-classical approaches for simulating nonadiabatic dynamics in molecular and nanoscale systems.^29,30^ Furthermore, semiclassical mapping dynamics based on the Meyer-Miller-Stock-Thoss (MMST) Hamiltonian^31,32^ have emerged as a robust tool for studying population dynamics across multiple electronic states. By representing multiple electronic states with classical mapping variables, advanced approaches such as the linearized semiclassical (LSC)^33–35^ and symmetrical quasiclassical (SQC)^36,37^ methods have been successfully applied. These methods provide a powerful framework for treating electronic population dynamics alongside nuclear motion, demonstrating that environmental and nuclear dynamical effects are crucial for accurate simulations.

Despite these methodological advances, direct all-atom (AA) nonadiabatic simulations remain computationally expensive.^29,38^ An AA nonadiabatic simulation requires tracking electronic information along many nuclear configurations, while the nuclear motion itself involves an immense number of degrees of freedom. In practice, this restricts direct atomistic simulations to spatial scales of a few nanometers or tens of thousands of atoms, limiting the accessible time scales and falling short of realistic OPV device sizes. A common workaround to bypass this bottleneck is the construction of effective model Hamiltonians. The recently developed multistate harmonic (MSH) model provides a formally consistent framework that generalizes the two-state spin-boson model to an arbitrary number of electronic states.^39–43^ By extracting energy-gap fluctuations from AA molecular dynamics (MD) and electronic structure calculations, the MSH model maps the solvent environment onto a globally shared harmonic bath. The MSH model has been demonstrated to faithfully reproduce AA nonadiabatic molecular dynamics for several realistic condensed-phase systems.^39,40,44^ A key feature underlying this capability is the preservation of the pairwise reorganization energies associated with all electronic transitions, which is achieved through the construction of the globally shared harmonic bath. The MSH model therefore retains the primary influence of environmental fluctuations on the electronic states while avoiding the explicit treatment of the full condensed-phase environment during the subsequent nonadiabatic dynamics simulations. Thus, the model retains the primary effect of the fluctuating environment on the electronic states while avoiding the explicit treatment of the full condensed-phase system at every step.

However, the MSH approach significantly reduces the total degrees of freedom at the level of the model Hamiltonian; it does not directly provide the explicit molecular motion of the actual system. For large OPV systems containing tens of thousands of atoms, the abstract effective normal-mode picture cannot tell us about the molecular mechanisms underlying energy conversion. Because processes such as exciton diffusion, multistep charge separation, and charge transport occur on larger spatial and temporal scales, the molecular representation itself must be simplified. Coarse-grained (CG) force fields address this by reducing the number of explicit molecular degrees of freedom.^45–47^ Many established CG parameterization methods, such as iterative Boltzmann inversion,^48,49^ force-matching,^50,51^ and relative entropy minimization,^52,53^ successfully reproduce static structural observables like radial distribution functions (RDFs). Unfortunately, the resulting CG potential is typically closer to a potential of mean force (PMF) than to a true potential energy surface (PES). While this distinction may be acceptable for structural sampling, it might be problematic for nonadiabatic dynamics involving CT phenomena.

For CT processes, local solute-environment interactions directly govern the energy-gap fluctuations. If a CG model overly smooths these short-range interactions, it washes out the critical energetic fluctuations required for accurate population transfer. Furthermore, it is a potential misconception that reproducing the energy gap statistics, i.e., the average energy gap and its variance (related to the reorganization energy), is sufficient to capture nonadiabatic molecular dynamics (NAMD). In reality, accurate kinetic and dynamical modeling requires the explicit dynamical information encoded in the energy-gap time correlation functions (TCFs).^39,41,54,55^ These TCFs dictate the spectral densities, which, alongside the simultaneous satisfaction of all pairwise reorganization energy constraints, govern the population dynamics. Over-smoothed CG models arguably destroy these TCFs and alter local atomistic friction. Omitting explicit atoms, as seen in united-atom (UA) models, often artificially accelerates molecular motions.^56–62^ While some CG methods attempt to patch this by applying generalized Langevin equations^63–68^ to add artificial friction, such mathematical manipulations do not correct the fundamentally inaccurate PES. A CG model used for nonadiabatic dynamics must therefore do more than reproduce structure; it must preserve the precise energetic and dynamical features that drive electronic transitions.

In this context, encoding vital nonbonded interaction information directly into the CG potential from the bottom up is essential. The Lennard-Jones static potential matching (LJSPM) method^69^ provides a highly effective semianalytical solution for this task. Unlike methods relying on long-term high-resolution presampling, LJSPM operates similarly to the restrained electrostatic potential fitting charges.^70^ It defines an electrostatic potential-like LJ static potential and optimizes the CG LJ parameters by matching this potential between the AA and CG representations. This method has been shown to accurately reproduce the atomistic LJ interaction energy landscape^69,71,72^ and capture key active site structures in molecular docking simulations.^73^ For CT dynamics, this energy-based parameterization is highly advantageous because it preserves the local solute-environment nonbonded interactions that directly shape the energy gap TCFs.

In this work, we combine the CG force fields derived from LJSPM with nonadiabatic semiclassical dynamics to construct a reduced-representation framework for photoinduced CT in the solution phase. To ensure the correct energetic alignments on the CG trajectory, we apply state-specific electronic energy corrections calculated via quantum chemistry and molecular dynamics. We utilize a prototypical OPV molecular triad, carotenoid-porphyrin-fullerene (CPC_60_), dissolved in explicit tetrahydrofuran (THF),^44,74–77^ alongside a perylene diimide (PDI) dimer dissolved in acetonitrile (ACN).^78,79^ By systematically defining solute/solvent systems with an AA/AA-level notation (i.e., all-atom for solute and all-atom for solvent), we test the extent to which fully atomistic NAMD can be reproduced at various CG levels. Furthermore, we investigate whether MSH models constructed from these CG force field simulations can preserve the vital energy gap TCFs and spectral densities necessary to match the all-atom NAMD benchmarks. To the best of our knowledge, a CG force field has never been successfully integrated with nonadiabatic dynamics for disordered condensed phases to reproduce the corresponding fully atomistic NAMD reference, making this bottom-up framework a uniquely powerful tool for bridging spatial scales in photochemical simulations.

The remainder of this paper is organized as follows. In Section 2, we present a brief overview of the multistate Hamiltonian at both the AA and CG levels, as parameterized via the LJSPM method, alongside the framework of the MSH model. Section 3 presents the simulation setup for the three-state CPC_60_ triad dissolved in THF and the four-state PDI dissolved in ACN, which serve as our benchmark systems for the NAMD. In Section 4, we report the results for both solution systems using descriptions on the fully atomistic, various CG levels, and the MSH models constructed from different MD simulations. Finally, we provide concluding remarks in Section 5.

## 2 Theory

### 2.1 All-atom Multistate Hamiltonian

To describe the nonadiabatic dynamics in disordered condensed phases, we begin by defining the general multistate Hamiltonian in the diabatic representation. For an *F*-state electronic system coupled to an *n*-atom environment, the total Hamiltonian is expressed as^44^

1 $$H=\sum_{i=1}^{n}\frac{p_{i}^{2}}{2m_{i}}+\sum_{\alpha ,\beta =1}^{F}V_{\alpha \beta }\left(R\right)\left|\alpha \right\rangle \left\langle \beta \right|$$

where |α⟩ denotes the α-th diabatic electronic state (α = 1,..., *F*), and **p**_*i*_^2^/(2*m*_*i*_) is the nuclear kinetic energy of the *i*-th atom with mass *m*_*i*_ (*i* = 1,..., *n*). The vectors **R** = (**r**_1_,..., **r**_*n*_) and **P** = (**p**_1_,..., **p**_*n*_) represent the 3*n*-dimensional atomic coordinates and momenta, respectively. The diagonal elements, *V*_*αα*_(**R**) = *V*_*α*_(**R**), correspond to the diabatic PESs for electronic state |*α*⟩. The off-diagonal elements, *V*_*αβ*_(**R**) = *Γ*_*αβ*_ (α ≠ β), represent the electronic couplings that drive the nonadiabatic population transitions between states |α⟩ and |β⟩, which are typically treated as constants within the Condon approximation.

In our fully AA reference and the foundational framework of the MSH model, the force field potential energy of the entire system on a certain state, including both the solute and the solvent, is given by the general Amber force field (GAFF)^80^

2 $$V^{\mathrm{AA}}=V_{\mathrm{bonded}}+V_{\mathrm{vdW}}+V_{\mathrm{elec}}$$

3 $$V_{\mathrm{bonded}}=\sum_{\mathrm{bonds}}K_{r}{\left(r-r_{0}\right)}^{2}+\sum_{\mathrm{angles}}K_{\theta }{\left(\theta -\theta_{0}\right)}^{2}+\sum_{\mathrm{dihedrals}}\sum_{k}K_{\phi ,k}\left[1+\cos\left(k\phi -\phi_{0,k}\right)\right]+\sum_{\mathrm{impropers}}K_{\varphi }\left[1-\cos\left(2\varphi \right)\right]$$

4 $$V_{\mathrm{vdW}}=\sum_{i<j}\epsilon_{\mathrm{ij}}\left[{\left(\frac{\sigma_{\mathrm{ij}}}{r_{\mathrm{ij}}}\right)}^{12}-2{\left(\frac{\sigma_{\mathrm{ij}}}{r_{\mathrm{ij}}}\right)}^{6}\right]$$

5 $$V_{\mathrm{elec}}=\sum_{i<j}\frac{1}{4\pi \epsilon_{0}}\frac{q_{i}q_{j}}{r_{\mathrm{ij}}}$$

Here, the bonded interaction *V*_bonded_ includes bond, angle, and dihedral terms, where *r*_0_, *θ*_0_ are bond and angle equilibrium parameters, *K*_*r*_, *K*_*θ*_, *K*_*ϕ*,*k*_, *K*_*φ*_ are the force constants for bond, angle, dihedral, and improper dihedral terms, *k* is the dihedral multiplicity and *ϕ*_0,*k*_ is the equilibrium torsional angle. The nonbonded interaction includes the van der Waals term *V*_vdW_ described by the pairwise Lennard-Jones (LJ) 12–6 potential^81^ with energy depth *ϵ*_*ij*_ and equilibrium distance *σ*_*ij*_ between atoms *i* and *j* that are separated by distance *r*_*ij*_ = |**r**_*i*_ – **r**_*j*_|,[Fn fn1] The Lorentz-Berthelot (LB) combination rule^82,83^ for the LJ parameters is given in the diameter style

6 $$\{\begin{array}{l} \sigma_{\mathrm{ij}}=\left(\sigma_{i}+\sigma_{j}\right)/2,\\ \epsilon_{\mathrm{ij}}=\sqrt{\epsilon_{i}\epsilon_{j}}. \end{array}$$

Additionally, the electrostatic or Coulomb term *V*_elec_ describes the interaction between all atomic pairs of partial charges *q*_*i*_ and *q*_*j*_ separated by distance *r*_*ij*_ with the vacuum dielectric constant *ϵ*_0_.

The diabatic PES for a given state |*α*⟩ of the solute is generally formulated by evaluating the interactions of the solute with its surrounding environment. Variations in *V*_*α*_(**R**) between different states predominantly arise from the differences in the atomic partial charges and the excitation energies of the solute. The total PES of the electronic state |*α*⟩ is written as^44,84^

7 $$V_{\alpha }\left(R\right)=V_{\alpha }^{\mathrm{AA}}\left(R\right)+W_{\alpha }\left(r_{\mathrm{solute}}\right)$$

where *V*_*α*_^AA^(**R**) is the force-field potential energy in eq 2 using the atomic charges of the solute on the *α*-th state {*q*_*i*_^(*α*)^}, and the state-specific energy correction *W*_*α*_(**r**_solute_) depends only on the solute geometry **r**_solute_, which can be determined by

8 $$W_{\alpha }\left(r_{\mathrm{solute}}\right)=E_{\alpha }\left(r_{\mathrm{solute}}\right)-V_{\alpha }^{\mathrm{AA}}\left(r_{\mathrm{solute}}\right)$$

Here, *E*_*α*_(**r**_solute_) is the excitation energy of the single solute molecule obtained from an electronic structure calculation. The term *V*_*α*_^AA^(**r**_solute_) is the force-field potential energy of the isolated solute molecule using the atomic partial charges obtained from the same electronic structure calculation. This energy correction removes the double-counted intramolecular contribution of the solute when the excitation energy of the solute molecule, obtained with a more accurate electronic structure calculation than the force field, is added to the overall potential energy.^44,84^ It is noted that if one were simulating adiabatic dynamics propagated on a single PES, the energy correction is not strictly necessary since a constant shift in potential energy will not change the curvature, and thus the forces that drive the dynamics. However, it is crucial for NAMD that occurs between different electronic states, as the relative energy differences directly affect the population transfer.

### 2.2 Bottom-Up Coarse-Grained Multistate Hamiltonian

To reduce the computational cost of simulating large-scale condensed-phase systems while retaining essential molecular motions, we project the AA representation onto a reduced CG representation. The total CG force field potential energy is defined similarly to traditional AA force fields, separating the interactions into bonded and nonbonded contributions^72^

9 $$V^{\mathrm{CG}}=V_{\mathrm{bonded}}^{\mathrm{CG}}+V_{\mathrm{vdw}}^{\mathrm{CG}}+V_{\mathrm{elec}}^{\mathrm{CG}}$$

10 $$V_{\mathrm{bonded}}^{\mathrm{CG}}=\sum_{\mathrm{bonds}}K_{r}{\left(r-r_{0}\right)}^{2}+\sum_{\mathrm{angles}}K_{\theta }{\left(\theta -\theta_{0}\right)}^{2}+\sum_{\mathrm{dihedrals}}\sum_{k=1}^{6}K_{\phi ,k}\left[1+\cos\left(k\phi -\phi_{0,k}\right)\right]+\sum_{\mathrm{impropers}}K_{\varphi }\left[1-\cos\left(2\varphi \right)\right]$$

11 $$V_{\mathrm{vdw}}^{\mathrm{CG}}=\sum_{i<j}\epsilon_{\mathrm{ij}}\left[{\left(\frac{\sigma_{\mathrm{ij}}}{r_{\mathrm{ij}}}\right)}^{12}-2{\left(\frac{\sigma_{\mathrm{ij}}}{r_{\mathrm{ij}}}\right)}^{6}\right]$$

12 $$V_{\mathrm{elec}}^{\mathrm{CG}}=\sum_{i<j}\frac{1}{4\pi \epsilon_{0}}\frac{q_{i}q_{j}}{r_{\mathrm{ij}}}$$

The parameterization of the CG force field follows a sequential bottom-up strategy. The bonded interactions (*V*_bonded_^CG^) are determined using the direct Boltzmann inversion (DBI) approach.^49,85^ By performing an isolated single-molecule simulation at the all-atom level, we extract the structural probability distributions, *P*(*x*), for the mapped CG internal coordinates (*x* = *r*, *θ*, *ϕ*, *φ* for bonds, angles, dihedrals, or improper dihedrals). The PMF is then obtained via the Boltzmann relation, *U*(*x*) = −*k*_*B*_ *T* ln *P*(*x*). Because CG mappings often result in broader or more complex structural distributions than their all-atom counterparts, these potentials are fitted to the higher-order polynomials specified in eq 10 (up to the 6th order for dihedrals) to accurately capture the structural flexibility of the CG molecule.^69^ For the electrostatic term (*V*_elec_^CG^), the effective partial charge *q*_*i*_ of a given CG bead is assigned simply as the scalar sum of the atomic partial charges of all constituent atoms within that mapped specific group.^69,72^

While DBI and direct charge summation effectively establish the molecular skeleton and long-range electrostatic interactions, the van der Waals term (*V*_vdw_^CG^) plays the most critical role in governing local solvent structure and short-range intermolecular packing. Traditional bottom-up methods parameterize these LJ interactions by matching free energy surfaces or RDFs.^49,86^ However, such trajectory-based matching often yields potentials of mean force that overly smooth the local interaction energies,^87–90^ a consequence that is detrimental to nonadiabatic dynamics, where precise local energy-gap fluctuations dictate population transfer. Additionally, free-energy-matching methods often fail to reproduce an accurate potential energy surface because CG models systematically underestimate conformational entropy contributions.^88^ To overcome this, we determine the LJ parameters (*ϵ*_*ij*_ and *σ*_*ij*_) using the LJSPM method, a bottom-up LJ parameterization strategy based on potential-energy-matching.^69^ Analogous to fitting partial charges to an electrostatic potential (ESP), LJSPM determines the CG LJ parameters by matching a three-dimensional static LJ potential field between the AA and CG representations. For a given structural configuration, the LJ static potential (LJSP) perceived by a probe particle *P* with an LJ radius σ_*P*_ is defined as^69^

13 $$\psi \left(r;\sigma_{P}\right)=\sum_{i}\sqrt{\epsilon_{i}}\min\left[{\left(\frac{\sigma_{P}+\sigma_{i}}{\left|r_{i}-r\right|}\right)}^{12}-2{\left(\frac{\sigma_{P}+\sigma_{i}}{\left|r_{i}-r\right|}\right)}^{6},1.0\right]$$

The truncation function min­[*x*, 1.0] is introduced to prevent the potential field from diverging in the highly repulsive core regions. The optimal CG parameters are obtained by minimizing the following error function between the AA and CG LJSP fields over the entire spatial domain.

14 $$\chi^{2}\left(\epsilon^{\mathrm{CG}},\sigma^{\mathrm{CG}};\sigma_{P}\right)=\int_{R^{3}}{\left|\psi^{\mathrm{CG}}\left(r,\epsilon^{\mathrm{CG}},\sigma^{\mathrm{CG}};\sigma_{P}\right)-\psi^{\mathrm{AA}}\left(r;\sigma_{P}\right)\right|}^{2}dr$$

Consequently, the resulting parameters σ^CG^(σ_*P*_), ϵ^CG^(σ_*P*_) would depend on the chosen *σ*_*P*_. The combination rule for the CG LJ parameters is given in the radius style^69^

15 $$\{\begin{array}{l} \sigma_{\mathrm{ij}}=\sigma_{i}\left(\sigma_{j}\right)+\sigma_{j}\left(\sigma_{i}\right),\\ \epsilon_{\mathrm{ij}}=\sqrt{\epsilon_{i}\left(\sigma_{j}\right)\epsilon_{j}\left(\sigma_{i}\right)}. \end{array}$$

However, the truncation in the strongly repulsive core can cause the original LJSPM method to systematically underestimate the CG particle radii (σ^CG^), leading to unphysically high mass densities and exaggerated local aggregation. To correct this without introducing arbitrary empirical repulsive terms, we apply the effective volume correction (EVC).^71^ The EVC approach mathematically defines an effective repulsive volume based on the zero-potential isosurface of the molecular groups. To this end, the AA effective volume of the *i*-th CG group of atoms (atomic set *N*_*i*_) for a probe particle *P* is defined as

16 $$V_{i\left(P\right)}^{\mathrm{AA}}=\int_{\sum_{j\in N_{i}}V_{\mathrm{jP}}^{\mathrm{LJ}}\left(r_{\mathrm{jP}}\right)>0}dr$$

Here, *V*_*jP*_^LJ^(*r*_*jP*_) is the LJ potential between the *j*-th atom and the probe *P*. We expect that the CG effective volume of the *i*-th CG bead, which is simply the spherical volume

17 $$V_{i\left(P\right)}^{\mathrm{CG}}=\frac{4\pi }{3}{\left(2^{-1/6}\sigma_{\mathrm{iP}}\right)}^{3}$$

to reproduce the value of *V*_*i*(*P*)_^AA^. In the EVC approach, the error function of the effective volumes between AA and CG for the particles *i* and *j* serving as probe particles of each other is defined as

18 $${\left(\chi_{\mathrm{EVC}}^{2}\right)}_{\mathrm{ij}}={\left(V_{j\left(i\right)}^{\mathrm{AA}}-V_{j\left(i\right)}^{\mathrm{CG}}\right)}^{2}+{\left(V_{i\left(j\right)}^{\mathrm{AA}}-V_{i\left(j\right)}^{\mathrm{CG}}\right)}^{2}$$

and minimizing this error function yields *V*_*i*(*j*)_^CG^ = *V*_*j*(*i*)_^CG^ = (*V*_*i*(*j*)_^AA^ + *V*_*j*(*i*)_^AA^)/2 and

19 $$\sigma_{\mathrm{ij}}^{\mathrm{CG}}={\left[\frac{3\sqrt{2}}{8\pi }\left(V_{i\left(j\right)}^{\mathrm{AA}}+V_{j\left(i\right)}^{\mathrm{AA}}\right)\right]}^{1/3}$$

However, for the entire system, minimizing the sum of errors across all pairs, i.e., ∑_*i* ≤ *j*_(*χ*_EVC_^2^)_*ij*_, is heavily dependent on off-diagonal matrix elements, and its solution requires an expensive iterative approach. A simplification for EVC is to only use the same kind of particle as the probe particle and thus minimize the sum of diagonal elements ∑_*i*_(*χ*_EVC_^2^)_*ii*_, which yields

20 $$\sigma_{\mathrm{ii}}^{\mathrm{EVC}}={\left(\frac{3\sqrt{2}}{4\pi }V_{i\left(i\right)}^{\mathrm{AA}}\right)}^{1/3}$$

21 $$\sigma_{i}^{\mathrm{EVC}}=\sigma_{\mathrm{ii}}^{\mathrm{EVC}}/2$$

With the CG particle radius parameters strictly constrained by the EVC, i.e., σ^CG^(σ_*P*_) = σ^EVC^, to ensure proper molecular volume and density, a restricted LJSPM optimization using eq 14 is performed to solve for the well depths (*ϵ* ^CG^(σ_*P*_)). This decoupled LJSPM-EVC parameterization ensures that the resulting CG potential closely reconstructs the true energetic topography of the nonbonded LJ interactions, directly preserving the magnitude and variance of the local solute-solvent fluctuations essential for accurate dynamics.

To construct the multistate CG Hamiltonian, it is equally necessary to incorporate the state-specific excitation energies to maintain the correct thermodynamic driving forces. The energy correction for the CG force field follows a similar procedure to the AA framework (eqs 7 and 8). The total diabatic potential energy and the corresponding correction term for the CG representation on state *α* are given by

22 $$V_{\alpha }\left(R^{\mathrm{CG}}\right)=V_{\alpha }^{\mathrm{CG}}\left(R^{\mathrm{CG}}\right)+W_{\alpha }\left(r_{\mathrm{solute}}^{\mathrm{CG}}\right)$$

23 $$W_{\alpha }\left(r_{\mathrm{solute}}^{\mathrm{CG}}\right)=E_{\alpha }\left(r_{\mathrm{solute}}\right)-V_{\alpha }^{\mathrm{CG}}\left(r_{\mathrm{solute}}^{\mathrm{CG}}\right)$$

Here, *E*_*α*_(**r**_solute_) represents the quantum mechanical excitation energy of the isolated solute molecule, which is calculated based on its underlying atomistic configuration **r**_solute_. The terms *V*_*α*_^CG^ (**R** ^CG^) and *V*_*α*_^CG^(**r**_solute_^CG^) denote the CG force field potential energy of the entire system (full configuration **R** ^CG^) and the isolated solute (solute configuration **r**_solute_^CG^), respectively, both evaluated using the partial charges assigned to the electronic state *α*. This formulation ensures that while the nuclear dynamics evolve on a reduced-resolution PES, the vertical energy gaps driving the charge transfer are rigorously anchored to high-level electronic structure calculations.

### 2.3 Multistate Harmonic Model Hamiltonian

While the AA and CG force fields provide explicit molecular coordinates for evaluating the total Hamiltonian, propagating nonadiabatic dynamics over tens of thousands of degrees of freedom remains computationally expensive. To achieve extremely efficient long-time dynamical propagation while retaining the essential environmental response, the explicit molecular systems can be mapped onto an effective MSH model.^39,40^

The MSH model replaces the complex explicit molecular environment with a globally shared bath of independent harmonic oscillators. Assuming the environmental fluctuations follow linear response theory, a widely valid approximation for solute-solvent interactions in liquid phases, the energy-gap fluctuations can be accurately represented by a harmonic bath.^91^ For the diabatic PESs *V*_*α*_(**R**) and *V*_*β*_(**R**) defined at either the AA or CG level, the vertical energy gap is defined as *U*_*αβ*_(**R**) = *V*_*α*_(**R**) – *V*_*β*_(**R**). The fluctuation of this energy gap relative to its ensemble average on a reference state (typically the initially excited state) is given by *δU*_*αβ*_(*t*) = *U*_*αβ*_(**R**_*t*_) – ⟨*U*_*αβ*_ ⟩ with **R**_*t*_ the nuclear configuration at time *t*.

As emphasized earlier, reproducing accurate nonadiabatic dynamics requires explicit dynamical information, not merely the static variance of the energy gap. This dynamical environmental response is encoded in the *F*(*F*–1) /2 energy-gap TCFs for an *F*-state system, and we define the TCF for the energy gap between states *α* and *β* as

24 $$C_{\mathrm{UU}}^{\left(\alpha \beta \right)}\left(t\right)=\left\langle \delta U_{\alpha \beta }\left(t\right)\delta U_{\alpha \beta }\left(0\right)\right\rangle$$

The pairwise reorganization energy *E*_*r*_^(*αβ*)^ is directly proportional to the variance of the energy gap, which corresponds to the zero-time TCF, via the following relation (where *k*_*B*_ is the Boltzmann constant and *T* is the temperature)^39,54^

25 $$E_{r}^{\left(\alpha \beta \right)}=\frac{C_{\mathrm{UU}}^{\left(\alpha \beta \right)}\left(0\right)}{2k_{B}T}$$

The frequency-dependent response of the environment driving the electronic transition is captured by transforming the TCF into the spectral density

26 $$J^{\left(\alpha \beta \right)}\left(\omega \right)=\frac{\omega }{4k_{B}T}\int_{0}^{\infty }dtC_{\mathrm{UU}}^{\left(\alpha \beta \right)}\left(t\right)\cos\left(\omega t\right)$$

The spectral density can then be discretized to yield a finite number *N*_*b*_ of distinct normal-mode frequencies {*ω*_*j*_|*j* = 1,..., *N*_*b*_}.^39,40,54,55^

The Hamiltonian of the MSH model is formulated by assigning a unique set of equilibrium displacements for each electronic state along a globally shared bath composed of these normal modes defined in an extended (*F* – 1) -dimensional subspace^39,40^

27 $$H^{\mathrm{MSH}}=\sum_{\alpha =1}^{F}\left(\sum_{d=1}^{F-1}\sum_{j=1}^{N_{b}}\frac{P_{d,j}^{2}}{2}+V_{\alpha }\left(R\right)\right)\left|\alpha \right\rangle \left\langle \alpha \right|+\sum_{\alpha \neq \beta }\Gamma_{\alpha \beta }\left|\alpha \right\rangle \left\langle \beta \right|$$

28 $$V_{\alpha }\left(R\right)=\sum_{d=1}^{F-1}\sum_{j=1}^{N_{b}}\frac{1}{2}\omega_{j}^{2}{\left(R_{d,j}-S_{j}^{\left(d,\alpha \right)}\right)}^{2}+\epsilon_{\alpha }$$

Here, *P*_*d*,*j*_, *R*_*d*,*j*_, and *ω*_*j*_ are the mass-weighted momentum, coordinate, and normal-mode frequency of the *j*-th normal mode of the bath in the *d*-th subspace, respectively (*j* = 1,..., *N*_*b*_; *d* = 1..., *F*–1). The total nuclear degrees of freedom are thus *N*_*n*_ = (*F* – 1) × *N*_*b*_. The parameter *ϵ*_*α*_ represents the potential energy minimum or the vertical shift of the *α*-th electronic state (*α* = 1,..., *F*) and can be obtained from the relation^39,40^

29 $$\epsilon_{\beta }-\epsilon_{\alpha }=\Delta E^{\left(\alpha \beta \right)}=-E_{r}^{\left(\alpha \beta \right)}-\left\langle U_{\alpha \beta }\right\rangle$$

The state-specific equilibrium shift component *S*_*j*_^(*d*,*α*)^ corresponds to the *j*-th mode in the *d* subspace for the *α*-th state. The nonvanishing shift components {*S*_*j*_^(*d*,*α*)^ |1 ≤ *d* < *α* ≤ *F*} are solved consistently to ensure that the reorganization energies between all *F*(*F* – 1)/2 pairs of electronic states strictly reproduce those obtained directly from the AA or CG molecular dynamics simulations (eq 25)^43^

30 $$E_{r}^{\left(\alpha \beta \right)}=\sum_{d=1}^{F-1}\sum_{j=1}^{N_{b}}\frac{1}{2}\omega_{j}^{2}{\left(S_{j}^{\left(d,\alpha \right)}-S_{j}^{\left(d,\beta \right)}\right)}^{2}$$

By doing so, MSH models built from realistic molecular dynamics faithfully preserve the critical dynamical energy-gap fluctuations that drive nonadiabatic dynamics while compressing the massive nuclear degrees of freedom into a highly efficient effective model Hamiltonian. Several successful applications have demonstrated the capability of the MSH model to accurately reproduce fully atomistic NAMD results in complex condensed-phase systems.^40,42,44,92^ Furthermore, this framework allows the pairwise reorganization energies to be used to unambiguously define the extent of correlated and anticorrelated bath effects.^93^

### 2.4 Nonadiabatic Semiclassical Mapping Dynamics

To propagate the nonadiabatic dynamics across the fully atomistic, coarse-grained, and MSH representation levels on an equal footing, we employ nonadiabatic semiclassical mapping dynamics. The MMST mapping framework offers a rigorous approach for transforming the discrete electronic state into continuous classical-like mapping variables, enabling the simultaneous propagation of both nuclear and electronic degrees of freedom. Within the MMST framework, the electronic operators can be expressed as^31,32^

31 $$\left|\alpha \right\rangle \left\langle \beta \right|\to M_{\alpha \beta }=\frac{1}{2\hbar }\left(q_{\alpha }-ip_{\alpha }\right)\left(q_{\beta }+ip_{\beta }\right)-\gamma \delta_{\alpha \beta }$$

where {*q*_*α*_, *p*_*α*_|*α* = 1,..., *F*} are the mapping variables. Here, γ is the zero-point energy (ZPE) parameter, which varies depending on the specific semiclassical method employed.^36,37^ A value of γ = 1/2 corresponds to the full quantum mechanical ZPE,^31,32^ which serves as our default value unless explicitly stated otherwise. Consequently, the *F*-state mapping Hamiltonian is expressed as

32 $$H\left(R,P,q,p\right)=\frac{P^{2}}{2}+\sum_{\alpha =1}^{F}V_{\alpha }\left(R\right)\left(\frac{1}{2\hbar }q_{\alpha }^{2}+\frac{1}{2\hbar }p_{\alpha }^{2}-\gamma \right)+\sum_{\alpha \neq \beta }^{F}V_{\alpha \beta }\left(R\right)\frac{1}{2\hbar }\left(q_{\alpha }-ip_{\alpha }\right)\left(q_{\beta }+ip_{\beta }\right)$$

The above mapping Hamiltonian is a function of both the coordinates and momenta (**R**, **P**) for *N* nuclear degrees of freedom and the electronic mapping variables (**q**, **p**) for *F* electronic states, thus the coupled dynamics of the entire system can be determined by integrating Hamilton’s equations of motion. The nuclear forces driving the trajectories are therefore defined by a weighted linear combination of the gradients of the diabatic PESs, with the instantaneous weighting factors governed by the evolving electronic mapping variables.^44^

In nonadiabatic dynamics, the primary quantity of interest is the electronic reduced density matrix (RDM), which is defined as the partial trace of the overall density matrix over the nuclear Hilbert space, i.e., *σ*(*t*) = Tr_*N*_[*ρ*(*t*)]. Assuming an initial condition of *ρ*(0) = *ρ*_*N*_(0) ⊗ |*μ*⟩⟨*μ*| that is the direct product of nuclear initial density *ρ*_*N*_(0) and the electronic population |*μ*⟩⟨*μ*|, the RDM elements at time *t* are given by

33 $$\begin{array}{ll} \sigma_{\alpha \beta }\left(t\right)={\mathrm{Tr}}_{N}{\mathrm{Tr}}_{e}\left[\rho_{N}\left(0\right)\left|\mu \right\rangle \left\langle \mu \right|e^{\mathrm{iHt}/\hbar }\left|\beta \right\rangle \left\langle \alpha \right|e^{-\mathrm{iHt}/\hbar }\right] & \left(33\right)\\ \approx {\left(\frac{1}{2\pi \hbar }\right)}^{N+F}\int dR_{0}dP_{0}dq_{0}dp_{0}{\left[\rho_{N}\left(0\right)\right]}_{W}\left(R_{0},P_{0}\right) & \\ \times \rho_{e}\left(q_{0},p_{0}\right){\left[\left|\mu \right\rangle \langle \mu |\right]}_{W}\left(q_{0},p_{0}\right){\left[\left|\beta \right\rangle \langle \alpha |\right]}_{W}\left(q_{t},p_{t}\right) & \left(34\right) \end{array}$$

Here, Tr_*N*_(·) and Tr_*e*_(·) are traces over the nuclear and electronic Hilbert spaces, respectively, [*ρ*_*N*_(0) ]_*W*_ represents the Wigner transform of the nuclear initial density, *ρ*_*e*_(**q**_0_, **p**_0_) is the initial distribution of the mapping variables, and [|*β*⟩⟨*α*|]_*W*_ denotes the Wigner transform of the electronic operators, (e.g., *M*_*αβ*_ as defined in eq 31).^33,35^

Different trajectory-based nonadiabatic mapping dynamics methods can be derived from this mapping framework by selecting specific values for the ZPE parameter γ and by adopting different statistical sampling techniques for the initial mapping variables, the Wigner transform of electronic operators, and the electronic population estimators. For instance, the traditional MF dynamics, the LSC mapping dynamics, and the SQC windowing approach can all be unified under this mathematical formulation. A detailed description of these specific dynamical methods is provided in Ref 44. In this study, these various dynamical approaches are applied consistently to the multistate Hamiltonians utilizing AA force fields, the CG force fields constructed via LJSPM-EVC method,^69,71^ and the effective MSH models.^39,40^ This consistent application allows us to systematically evaluate how well structural coarse-graining and subsequent abstract bath mappings preserve the nonadiabatic dynamics.

## 3 Simulation Details

### 3.1 Physical Systems

In this work, we investigate two prototypical physical systems: the carotenoid-porphyrin-fullerene (CPC_60_) triad dissolved in tetrahydrofuran (denoted as triad/THF)^44,74,75^ and a perylene diimide dimer dissolved in acetonitrile (denoted as PDI/ACN).^78,79^ Throughout this work, an AA/AA or CG/CG-style solute/solvent notation is used to label the specific representation level of each system. The various atomistic and coarse-grained representations for both physical systems are illustrated in Figure 1.

**Figure 1:**
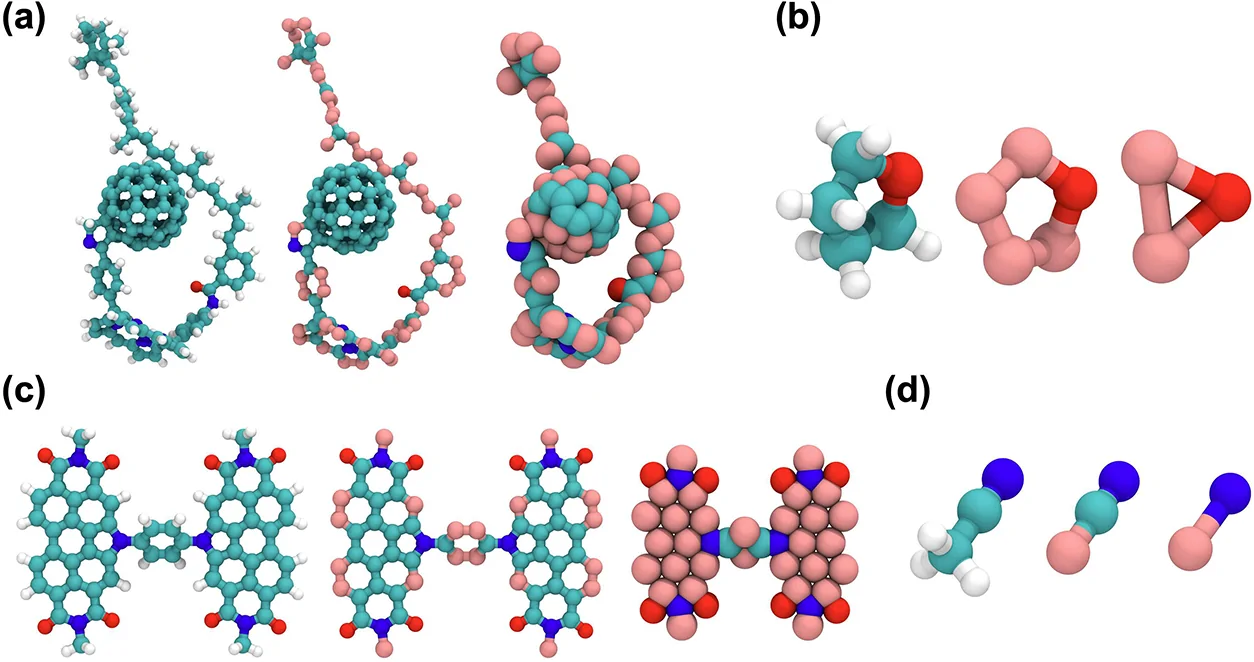
Various atomistic and coarse-grained (CG) representations of (a) carotenoid-porphyrin-fullerene (CPC_60_) triad on all-atom (AA), united-atom (UA) and 2-heavy-atom (2h) levels, (b) tetrahydrofuran (THF) on AA, UA, and 3-bead (3b) levels, (c) perylene diimide (PDI) dimer on AA, UA, and 2h levels, and (d) acetonitrile (ACN) on AA, UA, and 2-bead (2b) levels.

The triad/THF system is modeled as a three-state electronic system comprising the ground state, a locally excited ππ* state centered on the porphyrin moiety, and a charge transfer (CT) state representing electron transfer between the porphyrin and carotenoid, as shown in Figure S1. State-dependent electronic quantities were obtained from electronic-structure calculations at the ω*B97X-D/SV level with the range separation parameter of ω = 0.157,^94,95^ the electronic couplings between diabatic states were evaluated using the fragment charge difference (FCD) method.^96^ All electronic structure calculations were performed with Q-Chem 6.^97^ For both the AA and CG force fields, the relative alignments of the diabatic PESs are handled via the state-specific energy corrections defined in eqs 8 and 23. The excitation energies of the triad molecule for the ππ* and CT states are 1.5654 and 1.8081 eV, respectively, which are obtained with the Tamm–Dancoff approximation (TDA).^98^ The ground state PES has a zero energy correction, and all other excited states are shifted with respect to the ground state, as listed in Table 1. To extract the pairwise energy-gap TCFs and reorganization energies from the explicit MD simulations, equilibrium sampling every 5 fs over 6.7 × 10^6^ configurations was performed on the equilibrated ππ*-state PES.

**Table 1:** Energy Corrections *W*_α_ (in eV) for the CPC_60_ Triad and the PDI Dimer Systems, which are Dependent on the Solute CG Resolution and Independent of the Solvent Representation

triad			
state	AA	UA	2h
*W*_1_(ππ*)	1.728	1.757	0.104
*W*_2_(CT1)	0.184	1.828	1.342
*W*_3_(G)	0	0	0

PDI			
state	AA	UA	2h
*W*_1_(EX)	3.300	3.482	2.624
*W*_2_(CT1)	–0.408	–0.714	2.065
*W*_3_(CT2)	–0.572	–0.881	2.239
*W*_4_(G)	0	0	0

The PDI/ACN system is modeled as a four-state electronic system, including the ground state, an exciton (EX) state where the excitation is delocalized across the two PDI moieties, and two symmetric charge transfer states: CT1 (where electron transfer occurs from the left PDI moiety to the right PDI) and CT2 (where electron transfer occurs in the opposite direction) as shown in Figure S1. The corresponding electronic-structure calculations were carried out at the PBE/def2-SV­(P) level,^99,100^ using Q-Chem 6.^97^ The excitation energies of the PDI dimer for CT1, CT2, and EX states are 1.7198, 1.7207, and 2.0305 eV, respectively. Similar to the triad/THF system, the AA and CG force fields apply energy corrections to vertically shift the excited states relative to the ground state (Table 1). Equilibrium MD sampling every 5 fs over 8 × 10^6^ configurations to compute the pairwise energy-gap TCFs and reorganization energies for this system was performed on the EX-state PES.

To construct the CG multistate Hamiltonians, the CG force field parameters were derived using the XCG package.^69^ Starting from the AA force fields and the structures of the isolated solute and solvent molecules, CG representations were generated based on predefined mapping schemes described in the previous section. As explicitly shown in Figure 1, several specific CG resolutions are evaluated. For the triad/THF system, the CPC_60_ triad is represented at the AA, united-atom (UA), and 2-heavy-atom (2h) levels, while the THF solvent is represented at the AA, UA, and 3-bead (3b) levels. For the PDI/ACN system, the PDI dimer is represented at the AA, UA, and 2h levels, while the ACN solvent is represented at the AA, UA, and 2-bead (2b) levels.

The UA model groups each heavy atom and its directly bonded hydrogen atoms into a single CG bead, whereas the 2h model groups two adjacent heavy atoms and their bonded hydrogen atoms into one CG bead. The resulting CG force fields explicitly include bonded interactions (bonds, angles, and dihedrals) alongside the nonbonded van der Waals and electrostatic interactions for each CG species. The full CG topology is assembled by applying the combination rules between different CG species, as in the LJSPM-EVC method.^69,71^

The employed electronic couplings of the triad and PDI dimer systems across the AA and CG representation levels are summarized in Table 2. The electronic couplings of the triad were obtained directly from the FCD method with the electronic structure calculations. To obtain a significant population transfer in the PDI dimer system in the first few picoseconds such that AA NAMD is feasible, we modified the electronic coupling values based on the FCD values: the coupling between EX and CT1 was scaled up by 800 times, while the couplings between EX and CT2 and between CT1 and CT2 were scaled up by 50 times. Throughout this work, the dynamical simulations are performed within the Condon approximation, with the electronic couplings assumed to remain constant. This treatment is consistent with our simulation setup, in which the solute molecules are restrained using a harmonic force constant of 100 kcal mol^–1^ Å^–2^, thereby suppressing large-amplitude conformational changes of the solute that could otherwise lead to substantial variations in the electronic couplings. The use of the Condon approximation also allows us to focus specifically on the role of solvent reorganization in the nonadiabatic dynamics and to assess how faithfully this environmental contribution is retained upon coarse graining.

**Table 2:** Interstate Couplings *Γ*_*jk*_ (in eV) for the CPC_60_ Triad and the PDI Systems, which are System-specific and Independent of the Solute and Solvent CG Resolution

coupling	triad	PDI
*Γ*_12_	–3.72 × 10^–3^	7.44 × 10^–3^
*Γ*_13_		1.40 × 10^–2^
*Γ*_23_		1.65 × 10^–3^
*Γ*_*jG*_	0	0

Following the explicit MD simulations at the various AA and CG levels, the effective MSH models were parameterized by evaluating the instantaneous energy gaps along the chosen sampling trajectories. The energy-gap TCFs, reorganization energies, and spectral densities were computed using eqs 24 to 26. To represent the globally shared harmonic bath of the effective model, the spectral density was discretized into *N*_*b*_ = 200 nuclear normal mode frequencies. The MSH parameters, including the state-specific equilibrium shifts, were obtained by consistently satisfying the constraints of all pairwise reorganization energies. Unlike the explicit force fields, the MSH model establishes its energetic alignments using the energy minimum parameter *ϵ*_*α*_. These state energy minima were determined from the average energy gaps and the reorganization energies using eq 29. The sampling state, ππ* for triad/THF and EX for PDI/ACN, was chosen as the energetic reference state with its energy minimum set to zero, and the other electronic states were vertically shifted relative to it, as detailed in Table 3. The electronic couplings applied in the effective MSH models were identical to those used in the corresponding direct AA and CG multistate Hamiltonians.

**Table 3:** Energy Minima ϵ_α_ (in eV) of the Electronically Excited States in the MSH Models for Triad/THF and PDI/ACN Systems as Obtained from MD Simulations on Different Solute/Solvent CG Levels

triad/THF					
state	AA/AA	AA/UA	AA/3b	UA/UA	UA/3b
ϵ_1_(ππ*)	0	0	0	0	0
ϵ_2_(CT1)	–0.4095	–0.3967	–0.3123	–0.5541	–0.4228

PDI/ACN					
state	AA/AA	AA/UA	AA/2b	UA/UA	UA/2b
ϵ_1_(EX)	0	0	0	0	0
ϵ_2_(CT1)	–1.6643	–1.5974	–1.7266	–1.6108	–1.6739
ϵ_3_(CT2)	–1.6454	–1.5936	–1.7343	–1.5989	–1.6818

### 3.2 Nonadiabatic Dynamical Simulations

The triad/THF system contains one CPC_60_ triad molecule and 2700 THF molecules in a periodic cubic box of approximately 70 Å × 70 Å × 70 Å. The PDI/ACN system contains one PDI dimer and 1440 ACN molecules in a periodic cubic box of approximately 50 Å × 50 Å × 50 Å. Electrostatic interactions are treated using the particle mesh Ewald (PME) method,^101^ and the van der Waals cutoff is set to 9.0 Å. The SHAKE algorithm^102^ is used to constrain covalent bonds involving hydrogen atoms. The MD time step is chosen to be 1 fs. The systems are first equilibrated on the ground-state PES under the NPT ensemble at 300 K and 1.0 bar for 2 ns. A Langevin thermostat is applied with a collision frequency of 1.0 ps^–1^, and the pressure is controlled by a Monte Carlo barostat. Following this, the systems are further equilibrated under the NVT ensemble on the ground state for 1 ns.

After equilibration, a 20 ns ground-state NVT trajectory is generated for sampling. Initial nuclear positions and velocities are sampled every 0.2 ps from this trajectory to serve as the nuclear initial conditions for the NAMD simulations. During the NAMD production runs, structural configurations are saved every 200 fs to allow for structural analysis, such as computing time-dependent RDFs.

The NAMD simulations for all AA, CG, and MSH models are performed using various nonadiabatic mapping dynamics methods. These include the MF dynamics (γ = 0),^27^ SQC with a triangle window (γ = 1/3),^37^ LSC No. 1 (LSC1) and No. 2 (LSC2), along with their resolution-of-identity (RI) variants, i.e., RI-LSC1 and RI-LSC2 (γ = 1/2),^35,103^ and other mapping dynamics. The nonadiabatic trajectories are propagated using the QCDyn package^44^ interfaced with OpenMM.^104^ The nuclear time step is set to 1 fs.

NAMD simulations within the NVE ensemble are carried out on the full AA and CG Hamiltonians, which explicitly retain molecular details, as well as on the effective MSH models. The system is initialized by sampling nuclear configurations and velocities from the equilibrated ground state, followed by a vertical excitation to the initial excited state (ππ* for triad/THF, or EX for PDI/ACN) at time zero. For the initialization of the electronic mapping variables, LSC samples from a Gaussian distribution, SQC samples from a triangle window function, whereas MF requires no electronic sampling.^39^ The subsequent nonadiabatic dynamics are propagated strictly within the excited-state subspace: for the triad/THF system, this subspace is {ππ*, CT}, and for the PDI/ACN system, it is {EX, CT1, CT2}. To isolate the photoinduced CT dynamics on the excited-state manifold, the electronic couplings between the ground state and the excited states are set to zero. For the triad/THF system, each NAMD trajectory is propagated for 15 ps. The number of trajectories averaged to obtain the final RDM results depends on the dynamical method. Specifically, 1 × 10^4^ trajectories are used for MF, 5 × 10^4^ trajectories for all LSC variants, and 3.25 × 10^4^ trajectories for SQC. For the PDI/ACN system, the MF, LSC, and SQC trajectories are propagated for 5 ps, and 5 × 10^4^ trajectories are averaged for each method. All reported observables are obtained by averaging over these respective trajectory ensembles.

The overall workflow of this study, including the construction of the AA, CG, and corresponding MSH models and the subsequent comparison of their nonadiabatic dynamics, is summarized in Figure 2.

**Figure 2:**
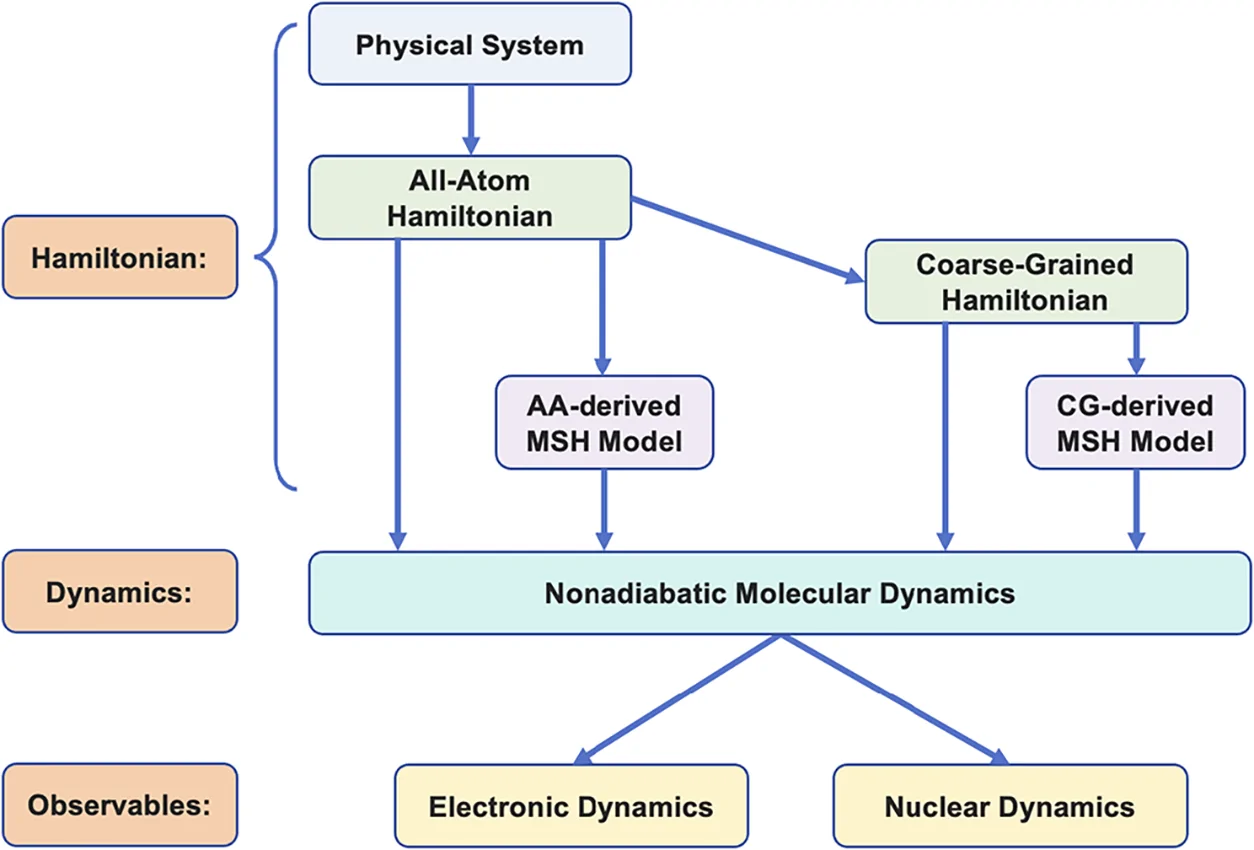
Flowchart summarizing the three main components of the present study: Hamiltonian, dynamics, and observables. CG Hamiltonians are constructed from the corresponding AA Hamiltonians, and multistate harmonic (MSH) model Hamiltonians are subsequently derived from both AA and CG representations. Nonadiabatic molecular dynamics (NAMD) simulations are then performed using the explicit-molecule AA and CG Hamiltonians as well as the corresponding effective MSH models. The resulting dynamics are characterized through electronic observables, such as population dynamics, and nuclear structural observables, such as time-dependent radial distribution functions.

## 4 Results and Discussion

### 4.1 Impact of Coarse-Graining on Reorganization Energy

The reorganization energy, *E*_*r*_, is a macroscopic measure of the environmental response to an electronic transition and is directly proportional to the variance of the energy-gap fluctuations. Accurately reproducing *E*_*r*_ is a fundamental prerequisite for any reduced-resolution model intended for nonadiabatic dynamics. The most critical reorganization energies are those between the relevant excited states (e.g., ππ* to CT in the triad, or EX to CT states in the PDI dimer), as these directly dictate the electronic-nuclear coupling for the nonadiabatic transitions during the simulation. Additionally, the *E*_*r*_ between the ground state and the initial optically bright state (G-ππ* or G-EX) is important because it relates to the initial nonequilibrium nuclear conditions following photoexcitation. Conversely, the reorganization energies between the ground state and the CT states are not explicitly traversed during the excited-state NAMD. Tables 4 and 5 present these pairwise reorganization energies evaluated across various solute/solvent representation levels for the triad/THF and PDI/ACN systems, respectively.

**Table 4:** Reorganization Energies *E*_*r*_ (in eV) for the Triad/THF System, as Obtained from MD Simulations on Different Solute/Solvent CG Levels

CG level	G-ππ*	G-CT	ππ*-CT
AA/AA	0.056	0.642	0.640
AA/UA	0.057	0.618	0.622
AA/3b	0.050	0.568	0.558
UA/UA	0.064	0.720	0.722
UA/3b	0.057	0.614	0.608
2h/UA	0.599	1.194	0.989

**Table 5:** Reorganization Energies *E*_*r*_ (in eV) for the PDI/ACN System, as Obtained from MD Simulations on Different Solute/Solvent CG Levels

CG level	G-EX	G-CT1	G-CT2	EX-CT1	EX-CT2	CT1-CT2
AA/AA	0.175	1.733	1.734	1.909	1.891	6.910
AA/UA	0.171	1.655	1.658	1.821	1.815	6.600
AA/2b	0.190	1.821	1.824	2.002	2.003	7.264
UA/UA	0.159	1.659	1.658	1.809	1.806	6.607
UA/2b	0.178	1.829	1.829	1.993	2.002	7.329
2h/UA	0.280	1.672	1.683	1.903	1.900	6.341

For the triad/THF system as shown in Table 4, the reference fully atomistic AA/AA simulation yields reorganization energies of 0.640 eV for the vital ππ*-CT transition and 0.056 eV for the G-ππ* transition. When the solute is maintained at the AA resolution while the solvent is coarse-grained (AA/UA and AA/3b), the critical ππ*-CT reorganization energies remain well-preserved, deviating by only 0.018 and 0.082 eV, respectively. The G-ππ* energy is similarly consistent. Coarse-graining the solute triad to the UA level (UA/UA and UA/3b) introduces slightly larger, yet acceptable, variations; the UA/UA and UA/3b models predict a ππ*-CT *E*_*r*_ of 0.722 and 0.608 eV, respectively. However, further reducing the solute resolution to the 2h level results in a severe breakdown of the energetic fluctuations. In the 2h/UA model, the *E*_*r*_ for the ππ*-CT transition is heavily overestimated at 0.989 eV, and the G-ππ* transition unphysically jumps an order of magnitude to 0.599 eV. This catastrophic deviation suggests that grouping two heavy atoms into a single bead for a highly conjugated solute like the CPC_60_ triad overly smooths the local intermolecular potential, destroying the delicate balance of atomic partial charges and nonbonded van der Waals interactions required to maintain proper energy-gap variances.

A similar trend is observed for the PDI/ACN system as shown in Table 5. The reference fully atomistic AA/AA simulation exhibits the expected symmetric environmental response due to the symmetric molecular structure of the PDI dimer (see Figure 1), with identical *E*_*r*_ values of approximately 1.90 eV for the EX-CT1 and EX-CT2 transitions, and a G-EX value of 0.175 eV. When the ACN solvent is coarse-grained to the UA or 2b levels while keeping the PDI dimer at the AA level, the reorganization energies remain highly consistent, and their deviations from the reference are on the level of 0.1 eV. For instance, the AA/UA model captures the overall magnitude and symmetry of the fluctuations, predicting *E*_*r*_ values of 1.821 eV for EX-CT1 and 0.171 eV for G-EX. Applying the UA mapping to the PDI dimer solute (UA/UA and UA/2b) also demonstrates excellent performance, preserving the EX-CT transition values within a narrow error margin of approximately 0.06 eV to 0.12 eV relative to the AA/AA benchmark. This indicates that the UA representation provides an optimal balance between computational efficiency and energetic accuracy for the PDI dimer. Conversely, further coarse-graining the solute to the 2h level (2h/UA) degrades the accuracy, noticeably increasing the G-EX reorganization energy to 0.280 eV, confirming that overly aggressive solute coarse-graining disrupts the local electronic-vibrational coupling.

These static energetic comparisons demonstrate that the solvent can be effectively coarse-grained using the LJSPM-EVC approach. The solvent coarse-graining retains the necessary solute-solvent interaction fluctuations, provided the solute is mapped at a sufficiently high resolution (AA or UA) to preserve the spatial distribution of its electrostatic and van der Waals features.

### 4.2 Effect of Coarse-Graining on Nonadiabatic Dynamics

We next examine how different CG levels affect the explicit-molecule nonadiabatic dynamics in both physical systems to determine whether they can effectively reproduce the electronic population dynamics obtained with the fully atomistic AA/AA benchmark. This comparison also addresses whether the previously observed differences in energy-gap fluctuations and reorganization energies strictly govern the nonadiabatic dynamic propagation. Figure 3 presents the nonadiabatic dynamics results for the triad/THF system, specifically the ππ* and CT state populations, represented across different CG levels and simulated with the MF, SQC, RI-LSC1, and RI-LSC2 dynamical methods. To facilitate direct comparison among the various models, a unified color scheme is adopted: the AA/AA reference is represented by a black curve; warm colors (e.g., brown for AA/UA, magenta for UA/UA) denote models with UA solvents; and cold colors (e.g., cyan for AA/3b, blue for UA/3b) denote models with the most coarse-grained solvent. The electronic states are distinguished by different line styles, with the initially populated ππ* state plotted as a solid line.

**Figure 3:**
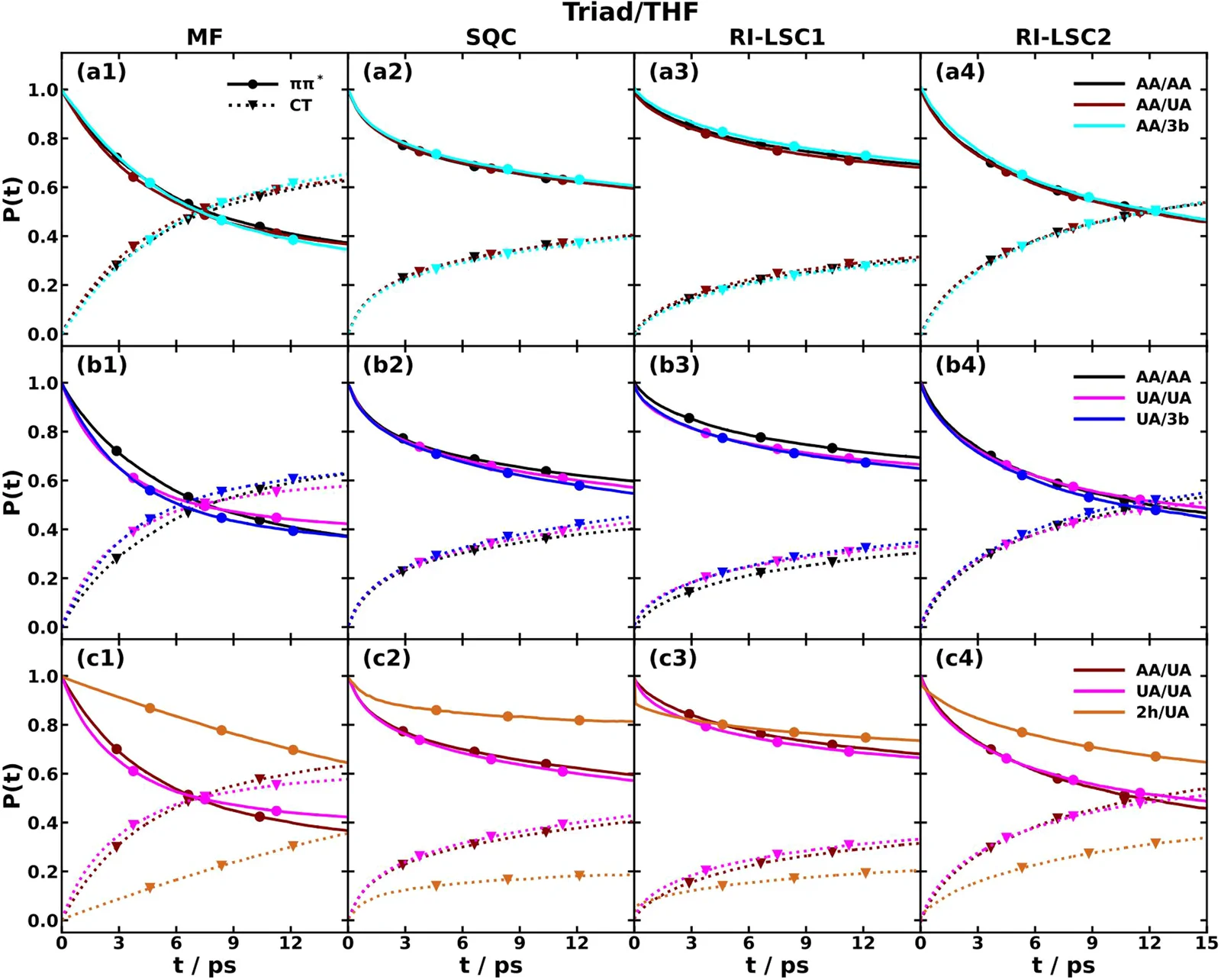
Comparison of nonadiabatic dynamics of the CPC_60_ triad/THF system represented on different CG levels and simulated with different dynamical methods, i.e., MF, SQC, RI-LSC1, and RI-LSC2 methods (from left to right). Rows represent comparison of CG levels for solute/solvent pairs: (a) Solute triad is fixed at AA level, while solvent THF is treated on AA, UA, and 3b levels. (b) Solute triad is treated on UA level, while solvent THF is treated on UA and 3b levels, compared with the fully atomistic reference. (c) Solute triad is treated on AA, UA, 2h levels, while solvent THF is fixed at UA level. Line styles represent the ππ* (solid with circles), CT (dotted with triangles) state populations.

Figure 3(a) demonstrates that when the solute is maintained at the AA level, the population dynamics of the AA/UA and AA/3b models completely coincide with the AA/AA benchmark generated by the same nonadiabatic dynamics method. Further coarse-graining the solute to the UA level yields similar success. Figure 3(b) shows the UA/UA and UA/3b models successfully reproduce the AA/AA reference, exhibiting only a minor population deviation of approximately 5%. It is noted that the variations between the different dynamical methods (where MF, RI-LSC2, SQC, and RI-LSC1 exhibit progressively decreasing decay rates of the initially populated state) are much more significant than the variations between the different CG levels. This indicates that these specific CG descriptions faithfully retain the local intermolecular interactions, the energy-gap fluctuations, the electronic-vibrational couplings, and the thermodynamic driving forces of the physical system.

Simplifying the solute triad further to beads of two heavy atoms (the 2h level) reveals a dramatic breakdown in the nonadiabatic dynamics. Figure 3(c) illustrates that the 2h/UA model underestimates the population decay significantly from the AA/AA reference, showing an electronic population error exceeding 20%. This dramatic failure indicates that the 2h representation of the highly conjugated solute severely misrepresents the local intermolecular interactions between the solute and the solvent. This large dynamical deviation traces directly back to the unfaithful energy-gap fluctuations and heavily overestimated reorganization energies previously identified for the 2h solute representation.

The PDI/ACN system exhibits highly consistent behavior. Figure 4 compares the explicit-molecule NAMD across different CG levels for the PDI/ACN system using the MF, SQC, RI-LSC1, and RI-LSC2 methods. Both the AA/UA and AA/2b models in Figure 4(a) and the UA/UA and UA/2b models in Figure 4(b) reproduce the AA/AA benchmark population dynamics exceptionally well, remaining within a 7% error limit. The population dynamics in the PDI/ACN system reveal an asymmetry in the charge transfer process. In the MF dynamics, the initially populated EX state transfers approximately 40% of its population to CT2 and only 18% to CT1 over the first 5 ps following photoexcitation. This broken symmetry mainly stems from the asymmetrical electronic couplings favoring the CT2 state in the studied PDI dimer geometry. Table 2 shows the EX-CT2 coupling is 1.40 × 10^–2^ eV, which is larger than the EX-CT1 coupling of 7.44 × 10^–3^ eV. Comparing the dynamical methods again confirms that MF, RI-LSC2, RI-LSC1, and SQC show varying decay rates, yet the viable CG models track their respective AA/AA benchmarks flawlessly. Applying the most aggressive 2h coarse-graining to the PDI dimer causes the EX state population to decay much more slowly than the reference. The 2h/UA model simulated with MF dynamics decays by only 0.0075% in the first 5 ps, whereas the AA/AA benchmark decays by over 60%. This failure to reproduce the electronic transition time scale confirms that the 2h representation loses the critical local intermolecular interactions and energy-gap fluctuations essential for NAMD.

**Figure 4:**
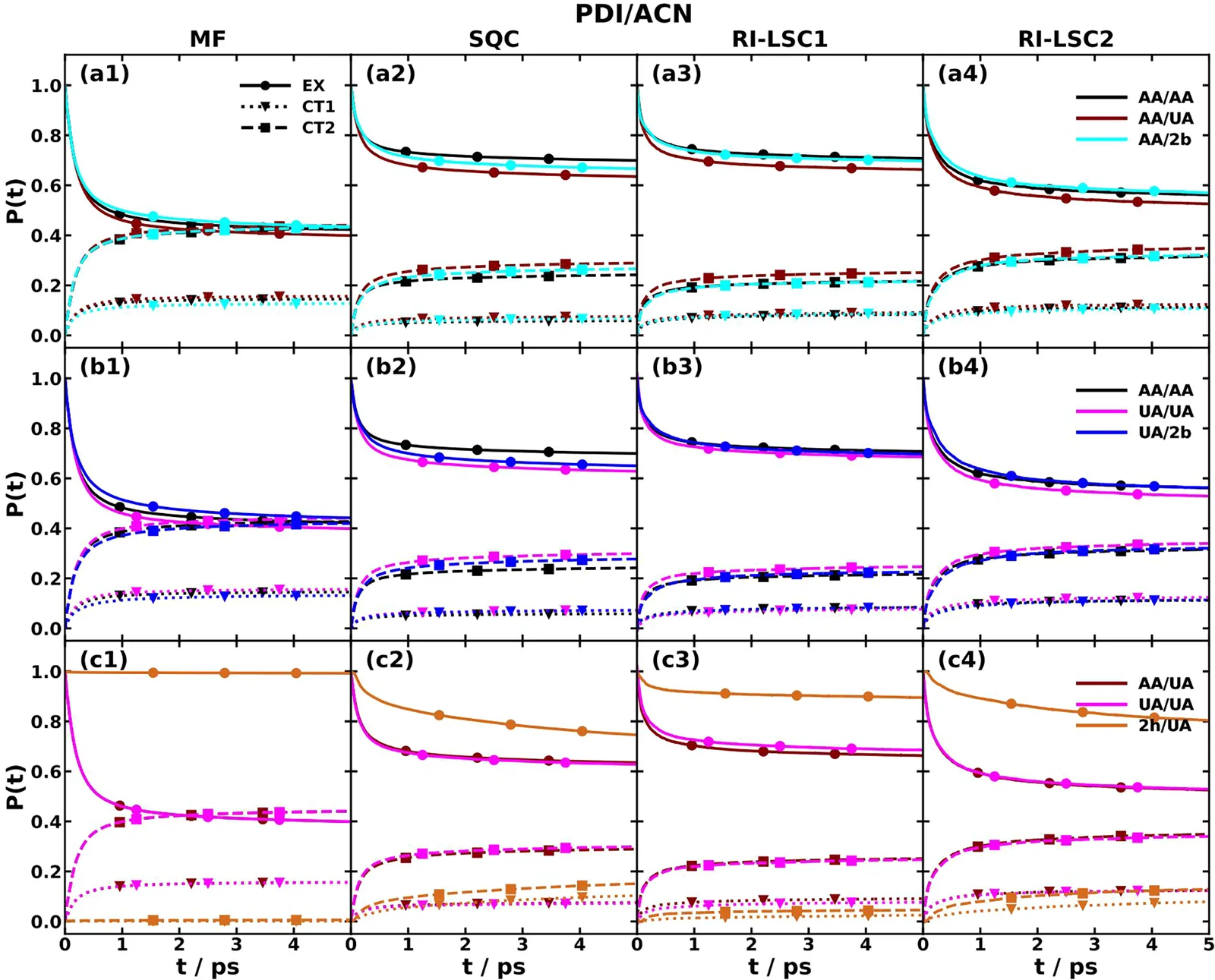
Comparison of nonadiabatic dynamics of the PDI/ACN system represented on different CG levels and simulated with different dynamical methods, i.e., MF, SQC, RI-LSC1, and RI-LSC2 methods (from left to right). Rows represent comparison of CG levels for solute/solvent system: (a) Solute PDI is fixed at AA level, while solvent ACN is treated on AA, UA, and 2b levels. (b) Solute PDI is treated on UA level, while solvent ACN is treated on UA and 2b levels, compared with the fully atomistic reference. (c) Solute PDI is treated on AA, UA, and 2h levels, while solvent ACN is fixed at UA level. Line styles represent the EX (solid with circles), CT1 (dotted with triangles), and CT2 (dashed with squares) state populations.

These NAMD comparisons across both physical systems establish a robust physical trend. Although the absolute numerical decay rates differ among the MF, SQC, RI-LSC1, and RI-LSC2 methods, their pattern of reproducing the AA/AA reference dynamics via coarse-graining remains highly consistent. Appropriate CG mapping of the solute (AA or UA) generally maintains population dynamics analogous to the AA/AA benchmark, while an excessively aggressive CG mapping (2h) leads to significant deviations. This consistency confirms that the findings are not artifacts of any particular propagation scheme, but accurately reflect the influence of the CG representation on the local intermolecular interactions and the electronic-vibrational coupling. We emphasize that for polar solvent molecules, the charge distribution, the local chemical environment, and the specific functional groups must dictate which atoms are merged into a CG bead. The CG partitioning should preserve the electrostatic and van der Waals environments to maintain the correct energy-gap fluctuations across multiple states. The 2b model of the ACN solvent succeeds precisely because it isolates the polar nitrogen atom as one bead and groups the remaining atoms into the second bead, whereas grouping the methyl and cyano groups as two beads fails. The design of CG mappings should therefore not be driven solely by the desire to reduce the total number of particles, but must carefully consider the local chemical environment to best preserve the underlying molecular interactions.

### 4.3 Explicit-molecule vs MSH Nonadiabatic Dynamics

In this section, we compare the nonadiabatic dynamics obtained with the explicit-molecule AA and CG Hamiltonians against the effective MSH models constructed from their corresponding MD simulations. Although the MSH models faithfully reproduce the reorganization energies and thermodynamic driving forces of the explicit-molecule equilibrium simulations, they neglect the anharmonicity of the PESs and aggressively reduce the spatial dimensions to 200 normal mode frequencies. Because the structural complexity of the explicit-molecule force fields is replaced by an abstract harmonic bath, the MSH model represents the most extreme form of coarse-graining tested in this work.

This dimensionality reduction raises two critical questions. First, how accurately can the MSH model reproduce the NAMD obtained with its corresponding explicit-molecule representation? Second, when projected into the MSH framework, do the minor energetic errors inherent to the valid CG force fields amplify or are preserved at a similar level? To answer these questions, we apply the same four dynamical methods to the MSH models for direct comparison with the explicit-molecule NAMD, strictly excluding the previously invalidated 2h solute representations.

Figure 5 presents the comparison of direct explicit-molecule NAMD against the MSH models for the triad/THF system. Overall, the MSH models reproduce the explicit-molecule NAMD results with a satisfactory error margin of approximately 5%. The MF and SQC methods exhibit slightly larger deviations than the RI-LSC1 and RI-LSC2 methods. Furthermore, the ππ* population decay and CT growth generated by the MSH models closely follow those from the corresponding explicit-molecule CG models and the AA/AA reference, with only small differences from the reference mainly appearing at longer times in MF dynamics. This indicates that projecting the atomistic anharmonic Hamiltonian onto a harmonic MSH representation does result in a slight loss of dynamical fidelity, but the main charge-transfer dynamics is preserved. A physical explanation is that explicit-molecule force fields possess a rugged energy landscape generating local friction and solvent caging effects that stabilize diffusive motions, which cannot be perfectly mimicked by purely harmonic continuous baths. Nevertheless, the deviation introduced by the MSH construction remains quite low. Achieving this level of accuracy is remarkable considering the MSH models discard tens of thousands of degrees of freedom and complex geometric interactions.

**Figure 5:**
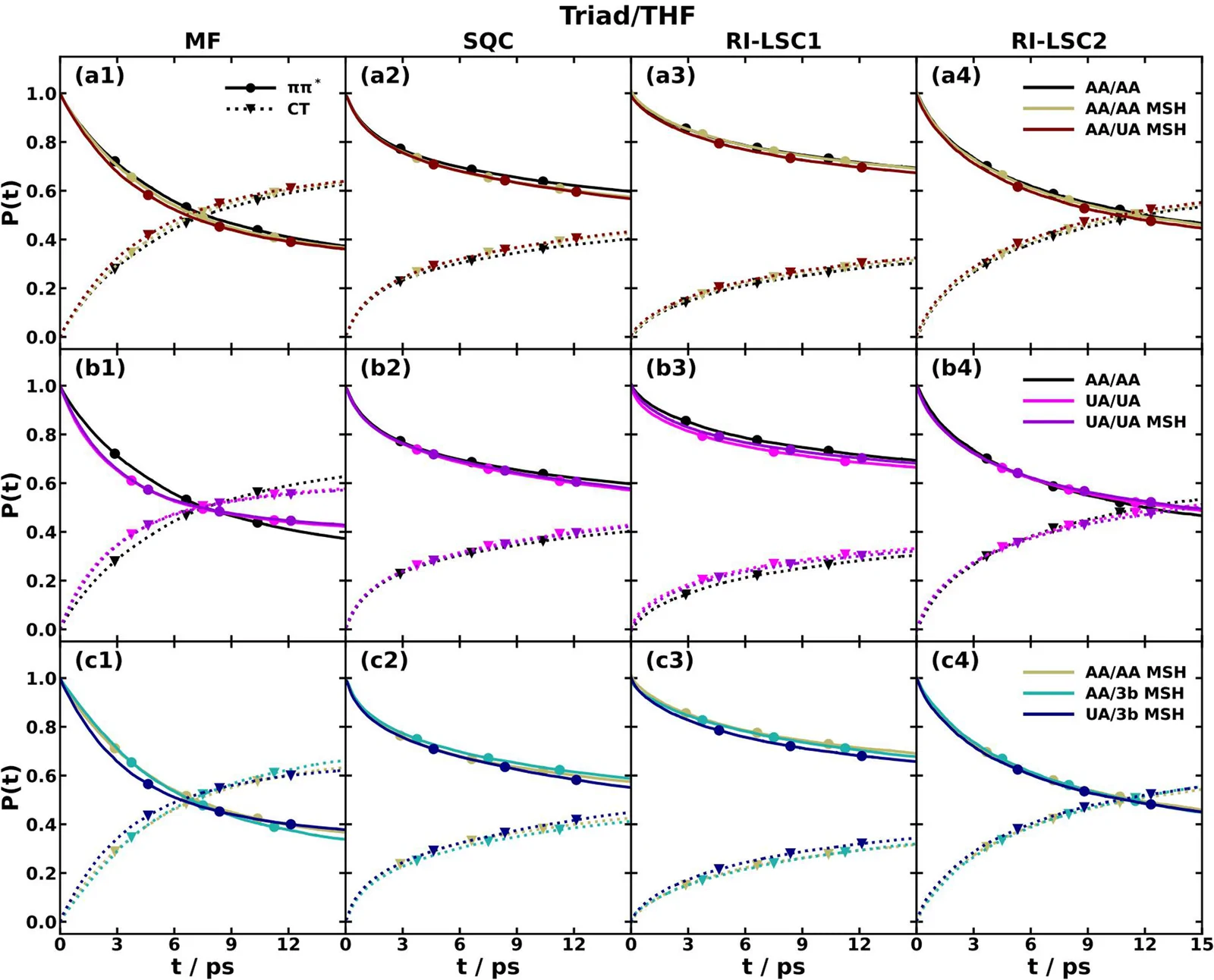
Comparison of nonadiabatic dynamics of the triad/THF system represented on different CG levels and the corresponding MSH models, simulated with different dynamical methods, i.e., MF, SQC, RI-LSC1, and RI-LSC2 methods (from left to right). Rows represent comparison of CG representations with molecular resolution against the MSH models constructed from certain CG levels: (a) MSH models constructed from AA/AA and AA/UA data, compared with the fully atomistic reference. (b) MSH model constructed from UA/UA data, compared with direct AA/AA and UA/UA results; (c) MSH models constructed from AA/AA, AA/3b, and UA/3b data. Line styles represent the ππ* (solid with circles), CT (dotted with triangles) state populations.

The PDI/ACN system exhibits a similar correspondence, as shown in Figure 6. The population dynamics obtained from the explicit-molecule NAMD closely align with those obtained from the corresponding MSH models for all four dynamical methods. In fact, the difference between the MSH model and the corresponding explicit-molecule simulations is minimal, compared with the more noticeable difference between explicit-molecule NAMD with various CG resolutions. For example, Figure 6(a),(b) exhibit nearly perfect agreement between the explicit-molecule NAMD and the corresponding MSH models on either AA/AA or UA/UA levels, but the population decay is faster in UA/UA than AA/AA, which is most apparent in SQC dynamics. This indicates that the effective energy-gap fluctuations used to parameterize the MSH Hamiltonians are consistently preserved such that the MSH models can reproduce the NAMD obtained directly with the corresponding explicit-molecule CG Hamiltonians.

**Figure 6:**
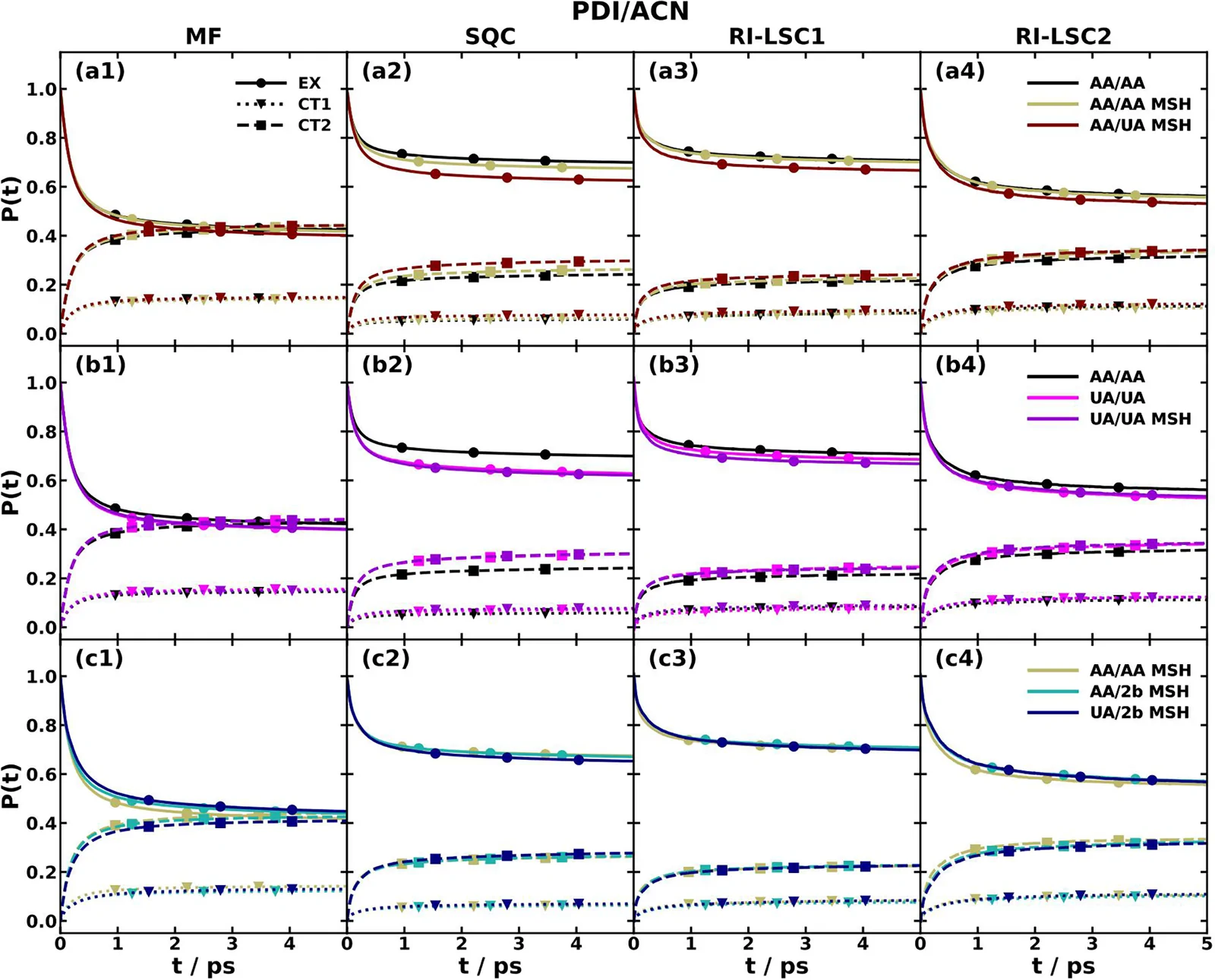
Comparison of nonadiabatic dynamics of the PDI/ACN system represented on different CG levels and the corresponding MSH models, simulated with different dynamical methods, i.e., MF, SQC, RI-LSC1, and RI-LSC2 methods (from left to right). Rows represent comparison of CG representations with molecular resolution against the MSH models constructed from certain CG levels: (a) MSH models constructed from AA/AA and AA/UA data, compared with the fully atomistic reference. (b) MSH model constructed from UA/UA data, compared with direct AA/AA and UA/UA results; (c) MSH models constructed from AA/AA, AA/2b, and UA/2b data. Line styles represent the EX (solid with circles), CT1 (dotted with triangles), and CT2 (dashed with squares) state populations.

These comparisons confirm that the true utility of a high-quality CG force field lies not only in accelerating explicit-molecule simulations but also in generating the accurate energy-gap TCFs required to parameterize effective model Hamiltonians. For both the triad/THF and PDI/ACN systems, the MSH dynamics derived from different CG resolutions remain highly consistent with the direct NAMD. This proves that the current CG parameterization successfully captured the essential electronic-nuclear coupling of the condensed-phase systems. The results from both the direct NAMD and the MSH levels collectively demonstrate that as long as a CG force field correctly preserves the key energy-gap fluctuations, reorganization energies, and interstate energy relations, it can stably reproduce the nonadiabatic behaviors of the fully atomistic benchmark while bypassing massive computational overhead.

### 4.4 Time Evolution of Structural Radial Distribution Functions

Nonadiabatic dynamics simulations capture not only the electronic RDM dynamics but also the explicit nuclear response of the solution. A primary advantage of performing explicit-molecule NAMD using AA and CG force fields is their ability to reveal real-time molecular structural dynamics alongside the photoinduced CT process. To quantify this, we analyze the time-dependent evolution of the RDFs, specifically examining the change Δ*g*(*r*) evaluated at specific time points during the NAMD relative to the equilibrium ground-state distribution at the moment of photoexcitation (*t* = 0).

The RDF differences for the triad/THF system are presented in Figure 7. These Δ*g*(*r*) profiles are averaged over the nonequilibrium MF trajectory ensemble at 1.0, 2.0, and 4.0 ps following photoexcitation. The left panels demonstrate that while the total density of the THF center of mass (COM) relative to the triad surface remains constant after photoexcitation, the distribution of the THF oxygen atoms exhibits a distinct increase in the 2–4 Å range and a corresponding decrease beyond 4 Å. This indicates a net reorientational motion of the THF molecules occurring without any bulk spatial translation or density change.^44,105^ A further spatial decomposition isolating the three moieties of the triad (right panels of Figure 7) provides a clearer molecular picture of this solvent reorientation. As the photoinduced CT progresses, the negatively charged oxygen atoms of THF show a strong, increasing preference toward the increasingly positively charged carotenoid donor moiety, evident from the rising Δ*g*(*r*) amplitude (blue lines). Conversely, the THF oxygen atoms actively turn away from the increasingly negatively charged fullerene acceptor moiety, resulting in the decreasing Δ*g*(*r*) amplitude (turquoise lines). The central porphyrin bridge (red lines) shows negligible preference change with respect to the solvent oxygen. Thus, the mechanistic picture reveals that nearby THF molecules undergo localized rotation, specifically orienting their negatively charged oxygen atoms toward the donor and away from the acceptor, without altering the net COM solvent density.

**Figure 7:**
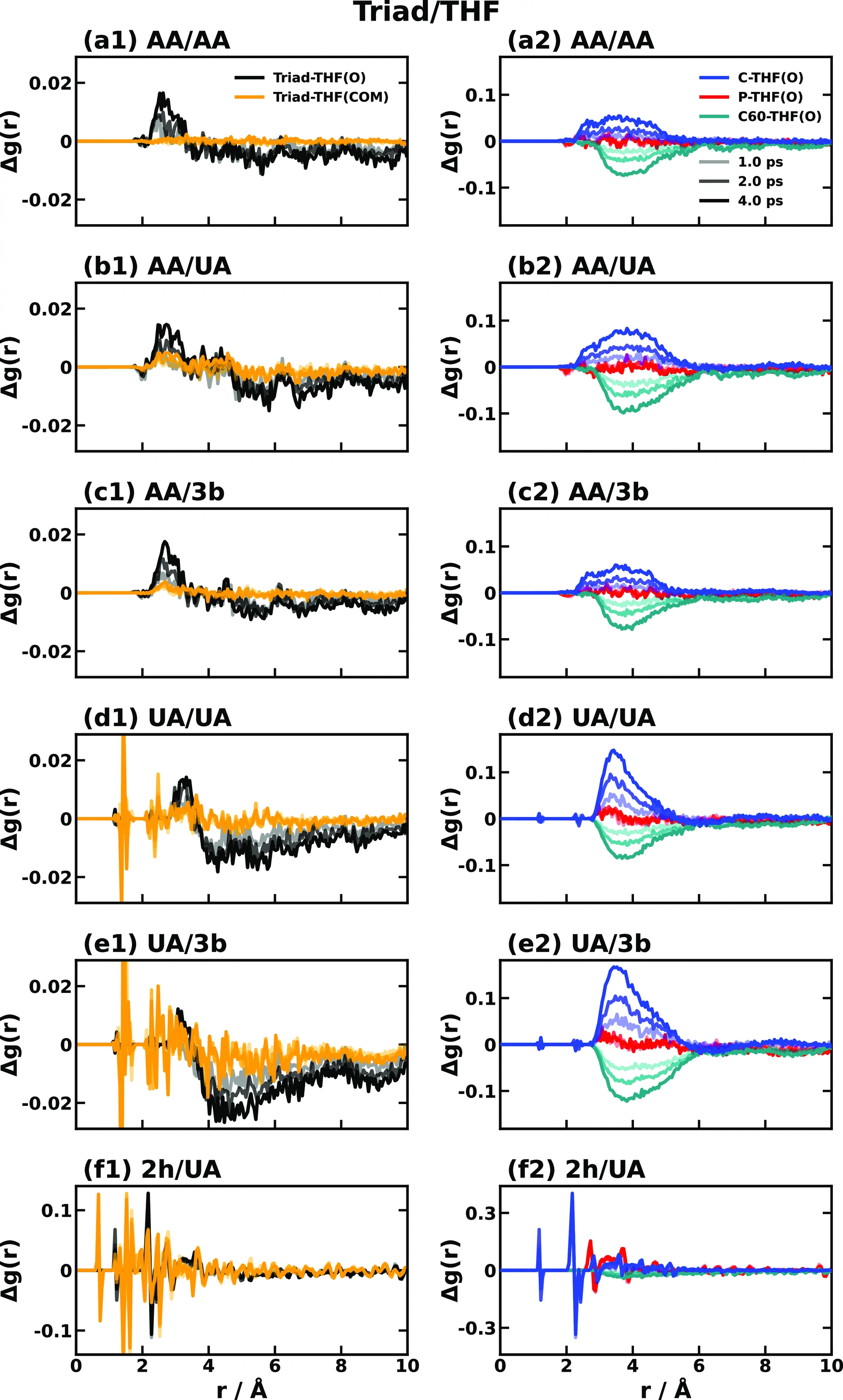
Time-dependent radial distribution function (RDF) Δ*g*(*r*) of triad/THF system between times 1.0, 2.0, and 4.0 ps with respect to time zero after the photoexcitation as obtained by MF dynamics. Left panels show RDF evolution for triad surface and the oxygen atom (O) of THF (black) and triad surface and center of mass (COM) of THF (orange); right panels show carotenoid (C, blue), porphyrin (P, red), and fullerene (C60, turquoise) moieties of the triad and the oxygen of THF contributions.

Comparing the time evolution of the RDFs across the different representation levels reveals that the AA/UA and AA/3b models capture behavior highly consistent with the AA/AA benchmark. When the solute is coarse-grained to the UA level (UA/UA and UA/3b), the RDFs preserve the overall physical trend but become noisier. Specifically, large fluctuations emerge in the 1–2 Å range. These artifacts can be attributed to the altered repulsive cores inherent to the UA mapping. Importantly, they do not interfere with the physically relevant solvation region beyond 2 Å. This indicates that while the broad energy-gap fluctuations are maintained, the fine details of the local solvent shell structure begin to degrade at the UA solute resolution. Finally, when the solute is aggressively coarse-grained to the 2h/UA level, both the total and moiety-specific RDFs degrade into significant noise. The critical mechanistic phenomenon of the donor attracting and the acceptor repelling the THF oxygen atoms is entirely lost. We can therefore conclude that the 2h representation is structurally insufficient to maintain a physically faithful molecular picture of the coupled solute-solvent dynamics during the photoinduced CT process.

For the PDI/ACN system as shown in Figure 8, the time-dependent RDFs obtained via MF dynamics demonstrate that the solvent response is also heavily localized to the first solvation shell. In the all-atom reference (Figure 8(a1)), the RDF between the PDI dimer and the ACN nitrogen atom (ACN­(N)) exhibits a distinct positive peak around 3 to 4 Å, whereas the change relative to the ACN center of mass is much smaller. This confirms that the ACN response is predominantly reorientational, accompanied by only a minor change in local number density. To examine the local rearrangements, we decompose the RDFs into the three moieties of the PDI dimer: the bridging benzene (B) in Figure 8(a2), and the left PDI (L) and the right PDI (R) in Figure 8(a3). At 0.2, 1.0, and 4.0 ps, the B-ACN­(N) and R-ACN­(N) pairs exhibit clear positive Δ*g*(*r*) changes, while the L-ACN­(N) pair displays a negative change over a similar spatial range. This indicates that following photoexcitation, the electronegative ACN nitrogen atoms become enriched near the bridge and right PDI regions, especially the bridge region, as evidenced by the large amplitude increase in its Δ*g*(*r*). This asymmetric solvation directly correlates with the electronic population dynamics, as the CT2 state (where the left PDI becomes more negatively charged) is populated approximately three times as much as the symmetrically opposite CT1 state.

**Figure 8:**
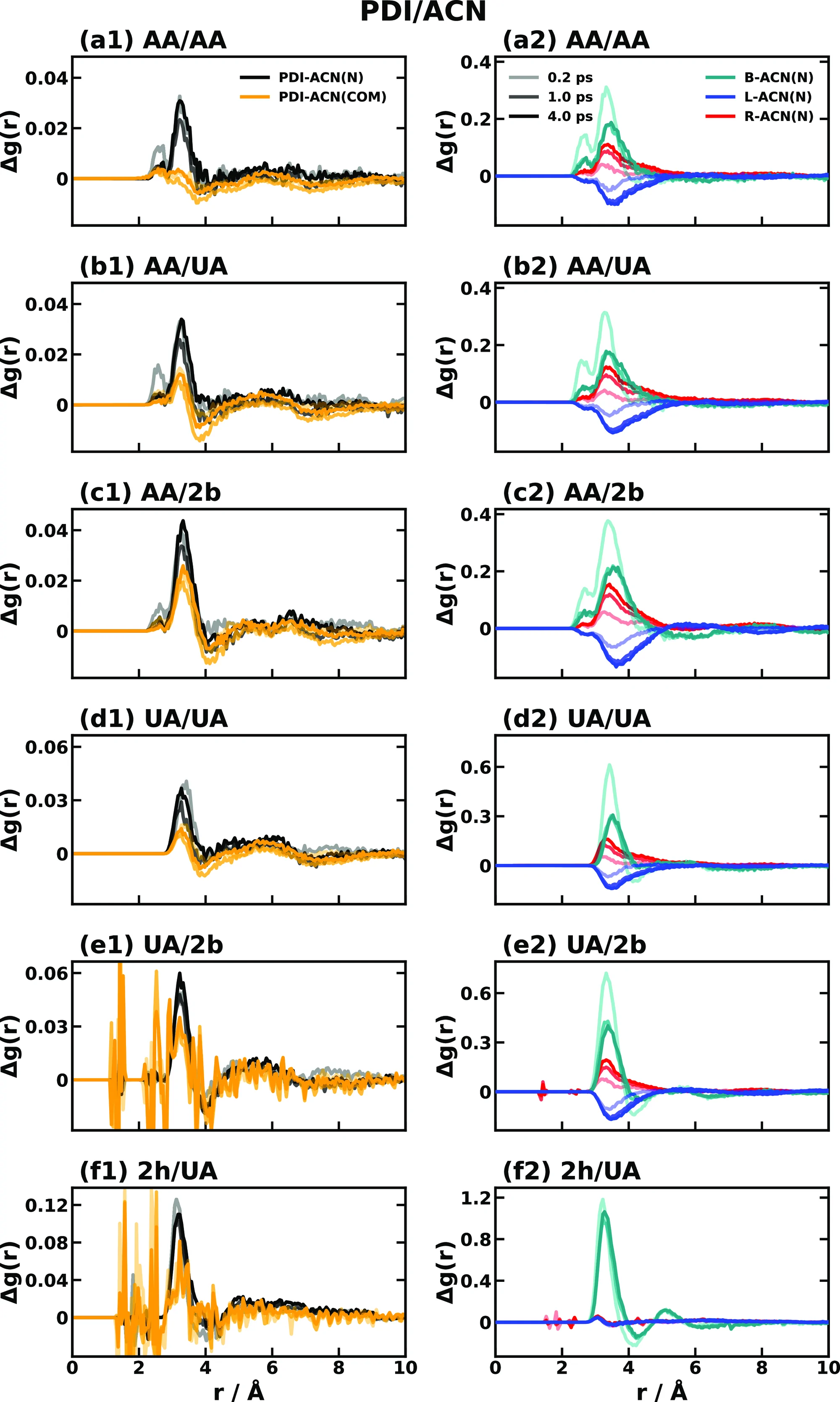
Time-dependent radial distribution function (RDF) Δ*g*(*r*) of PDI/ACN system between times 0.2, 1.0, and 4.0 ps with respect to time zero after the photoexcitation as obtained by MF dynamics. Left panels show RDF evolution for PDI dimer and the nitrogen atom (N) of ACN (black) and PDI dimer and the center of mass (COM) of ACN (orange); right panels show bridge (B, turquoise), left PDI (L, blue), and right PDI (R, red) moieties of the PDI dimer and the nitrogen of ACN contributions.

However, this fragment-dependent response cannot be fully explained by the static charge differences between the CT2 and EX states (see Table S2). For the left (L) and right (R) PDI moieties, the simple electrostatic argument holds: the L region becomes more negatively charged upon transitioning to CT2, driving the depletion of ACN­(N), while the R region becomes more positively charged, driving the local enrichment. In contrast, the bridge (B) region also becomes less positively charged upon the EX to CT2 transition, which would classically predict a depletion of ACN­(N). Yet, the time-dependent RDF reveals a localized enhancement. This anomaly arises from nonequilibrium dynamics. Immediately following the vertical excitation, the ACN­(N) atoms become highly enriched near the bridge region while the system remains predominantly on the EX state surface. This is corroborated by the significantly increased B-ACN­(N) RDF from the equilibrium ground state to the EX state, as shown in Figure S2­(b). As the population subsequently transfers from the EX state to the CT2 state, the overall attraction to the bridge decreases from its EX-state peak, gradually approaching the lower CT2 distribution, which more closely resembles the ground state. These dual time scales of the B-ACN­(N) interaction are directly evident in the time evolution of the Δ*g*(*r*) peak height shown in Figure S2­(e). An initial rapid rise occurs within the first 0.16 ps due to the abrupt vertical photoexcitation to the EX state, as also detailed in the short-time profiles in Figure S2­(e,f). This is followed by a decay until 0.40 ps, after which the peak value of Δ*g*(*r*) stabilizes at around 0.19, accompanying the gradual photoinduced CT to the predominantly populated CT2 state. Therefore, the observed positive Δ*g*(*r*) for B-ACN­(N) in the early picoseconds represents a transient, nonequilibrium solvent rearrangement strongly driven by the initial EX state geometry, rather than an equilibrated CT2-state distribution.

Varying the degree of coarse-graining significantly affects the ability to reproduce these complex structural responses. The AA/UA, AA/2b, and UA/UA models preserve the primary characteristics of the AA/AA benchmark exceptionally well. The first-shell peak positions are consistent, and the qualitative behavior of B/L enhancement versus R depletion remains intact. This confirms that a moderate degree of coarse-graining successfully captures the key solvent responses driving NAMD. Among these, the AA/UA, AA/2b, and UA/UA curves are the most stable and align closest with the fully atomistic reference. Further coarsening to the UA/2b level introduces noticeable noise in the short-range region of the total RDF, particularly in the COM correlation curve, indicating that the balance between local packing and molecular orientation begins to degrade. The most severe deviation again occurs in the 2h/UA model. The structurally continuous first-shell response is completely disrupted by large short-range oscillations, demonstrating that excessive coarse-graining cannot reliably reproduce the dynamic solvent rearrangement induced by local charge redistribution.

Across both physical systems, the time-dependent explicit-molecule RDFs confirm that the dominant structural response driving the photochemistry is the localized reorientational adjustment of the first solvent shell, rather than a bulk translational shift in solvent density. Regarding the impact of structural mapping, moderate CG resolutions (such as AA/UA, AA/solvent-bead, and UA/UA) effectively maintain the primary peak positions, amplitudes, and fragment-specific trends established by the AA/AA benchmarks. Conversely, aggressive 2h coarse-graining consistently introduces strong short-range artifacts, distorts the local solvation shell, and obliterates the fragment-specific solvation mechanisms essential for accurate nonadiabatic dynamics.

### 4.5 Computational Efficiency of Coarse-Grained Models

Finally, we briefly assess the computational cost and efficiency gains associated with the different CG representation levels. Because the production NAMD simulations were performed across various CPU-GPU platforms, we conducted an independent, strictly controlled benchmark on a single unified platform (NVIDIA RTX 3080 GPU) to provide a direct apples-to-apples comparison.

Table S4 summarizes the wall times for the NAMD simulations using the MF method, where 1250 independent trajectories were propagated for 15 ps for the triad/THF system and 5 ps for the PDI/ACN system. For the triad/THF system, coarse-graining the solvent from AA/AA (35,307 particles) to AA/3b (8,307 particles) reduces the wall time from 52,265 s to 20,763 s, achieving a speedup of approximately 2.5 times. A similar trend is observed for the PDI/ACN system, where moving from AA/AA to AA/2b reduces the particle count by roughly 66% and yields a measurable reduction in propagation time. However, the reduction in NAMD wall time does not scale linearly with the reduction in particle number. This sublinear scaling occurs because several computational components impose fixed or semi-fixed costs. These include GPU communication overhead, neighbor-list updates, and input/output operations.

Furthermore, it is important to note that the explicit benchmark systems evaluated here were intentionally constrained to relatively small sizes (roughly 50 to 70 Å cubic boxes) specifically to allow the fully atomistic AA/AA NAMD simulations to serve as a computationally feasible reference. Consequently, these small system sizes do not demonstrate the full acceleration capacity of the CG models. To illustrate the true computational scaling potential of these CG representations for macroscopic condensed-phase systems, we evaluated pure classical MD wall times for much larger solvent boxes. As shown in Table S5, standard MD production runs (50 ps) were performed for PDI/ACN systems containing one PDI molecule and different numbers of ACN molecules, i.e., 14,400 and 144,000. For the massive 144,000 ACN system, reducing the resolution from AA (864,100 particles) to the 2b CG level (288,100 particles) cuts the wall time from 612 s down to 284 s. Without the overhead of the electronic state propagation, the classical MD simulation achieves a speedup of roughly 2.2 times, which aligns much closer to the theoretical scaling expected from the particle reduction.

These scaling benchmarks confirm that while fixed algorithmic overheads buffer the speedups in small-scale nonadiabatic dynamics, the LJSPM-EVC coarse-grained force fields provide substantial computational savings. This makes the CG methodologies exceptionally valuable for extending explicit-molecule NAMD simulations to the massive length and time scales typical of realistic optoelectronic devices and complex biological environments.

## 5 Concluding Remarks

In this work, we systematically investigated the impact of coarse-graining on the photoinduced CT dynamics of condensed-phase systems. Utilizing the bottom-up LJSPM-EVC parameterization framework, we constructed a hierarchy of CG force fields, ranging from fully atomistic to united-atom and multi-heavy-atom bead models, for two prototypical photochemical systems: a CPC_60_ triad in THF and a perylene diimide dimer in ACN. By employing various semiclassical mapping dynamics (specifically MF, SQC, RI-LSC1, and RI-LSC2), we propagated the photoinduced charge transfer across three distinct representational tiers: fully atomistic explicit-molecule models, reduced-resolution CG explicit-molecule models, and highly reduced effective MSH models.

Our comprehensive evaluations of reorganization energies, electronic population dynamics, and time-dependent RDFs reveal a clear boundary for the physical fidelity of CG mappings in nonadiabatic processes. We demonstrated that the solvent environment can be aggressively coarse-grained (e.g., 3-bead THF or 2-bead ACN) while maintaining excellent agreement with the fully atomistic benchmark. Similarly, mapping the solute at the UA resolution robustly preserves the critical local intermolecular interactions, the asymmetric solvent reorientation, and the energy-gap fluctuations that drive the electronic transitions. However, further simplifying the highly conjugated solutes to a 2-heavy-atom representation drastically distorts the local electrostatic and van der Waals environments, leading to unphysical reorganization energies, the loss of specific solvation shell structures, and a severe breakdown in the resulting population dynamics.

Ultimately, our results confirm that high-quality, physically motivated CG force fields not only accelerate explicit-molecule NAMD but also serve as highly reliable foundations for parameterizing effective model Hamiltonians. The MSH models derived from the structurally valid CG simulations successfully reproduced the nonadiabatic dynamical behaviors of the explicit atomistic references while operating with a drastically reduced number of degrees of freedom. Combined with the substantial speedups observed in our computational scaling benchmarks, this unified multilevel approach provides a highly efficient and rigorously validated methodology. By bridging LJSPM-EVC coarse-graining, semiclassical mapping dynamics, and MSH projection, this framework paves the way for simulating long-time nonadiabatic dynamics in massive, realistic environments, such as complex organic photovoltaics and biological light-harvesting architectures.^106–108^

## Supplementary Material



## Data Availability

The data and scripts that support the findings of this study are available within the published article, the Supporting Information, and at https://github.com/xiangsunlab/CG-NAMD/.

## Footnotes

1 Note the definition of *σ*_*ij*_ in eq 4 is different from conventional LJ potential given by *V*_LJ_(*r*) = 4*ϵ*[(*σ*/*r*)^12^ −(*σ*/*r*)^6^] and *σ*_*ij*_ = 2^1/6^ *σ* is the equilibrium distance.

## Notes

Natural transition orbitals and state-specific moiety charges of the triad and PDI dimer systems, numerical simulation parameters of dynamics, computational efficiency benchmarks, and supplementary figures of the nonadiabatic population dynamics and time-dependent radial distribution functions across different semiclassical mapping methods (PDF)
