# Supplementary material for: Coarse-Grained Nonadiabatic Dynamics in Disordered Condensed Phases
Source: J Chem Theory Comput. 2026 Sep 4;22(18):9311–31. doi: 10.1021/acs.jctc.6c01363 (PMC13602924; doi:10.1021/acs.jctc.6c01363)
Supplement: Supplementary file 1 [file ct6c01363_si_001.pdf]

Supporting Information:
Coarse-Grained Nonadiabatic Dynamics in Disordered Condensed Phases

Zailing Song,[1,2,3] Zengkui Liu,[1,2,3] Fei Xia,[4,3] Yuwei Zhang,[5,a)] and Xiang Sun[1,2,3,b)]
[1)]*Division of Arts and Sciences, NYU Shanghai, 567 West Yangsi Road, Shanghai 200124, China*
[2)]*Department of Chemistry, New York University, New York, New York 10003, United States*
[3)]*NYU-ECNU Center for Computational Chemistry at NYU Shanghai, 3663 Zhongshan Road North, Shanghai 200062, China*
[4)]*School of Chemistry and Molecular Engineering, East China Normal University, Shanghai 200062, China*
[5)]*School of Chemistry and Materials Science, Nanjing Normal University, Nanjing 210000, China*

I. ELECTRONIC STATES OF THE INVESTIGATED MOLECULAR SYSTEMS

We provide detailed electronic structure information for the two molecular systems investigated in this work: the carotenoid-porphyrin-fullerene (CPC_{60}) triad[1] and the perylene diimide (PDI) dimer.[2] To visually characterize the nature of the relevant electronic transitions, Fig. S1 illustrates the natural transition orbitals (NTOs) associated with the locally excited ($\pi\pi^*$ and EX) and charge transfer (CT) states for both molecules. Tables S1 and S2 summarize the total charge of each molecular moiety for the triad and PDI dimer, respectively.

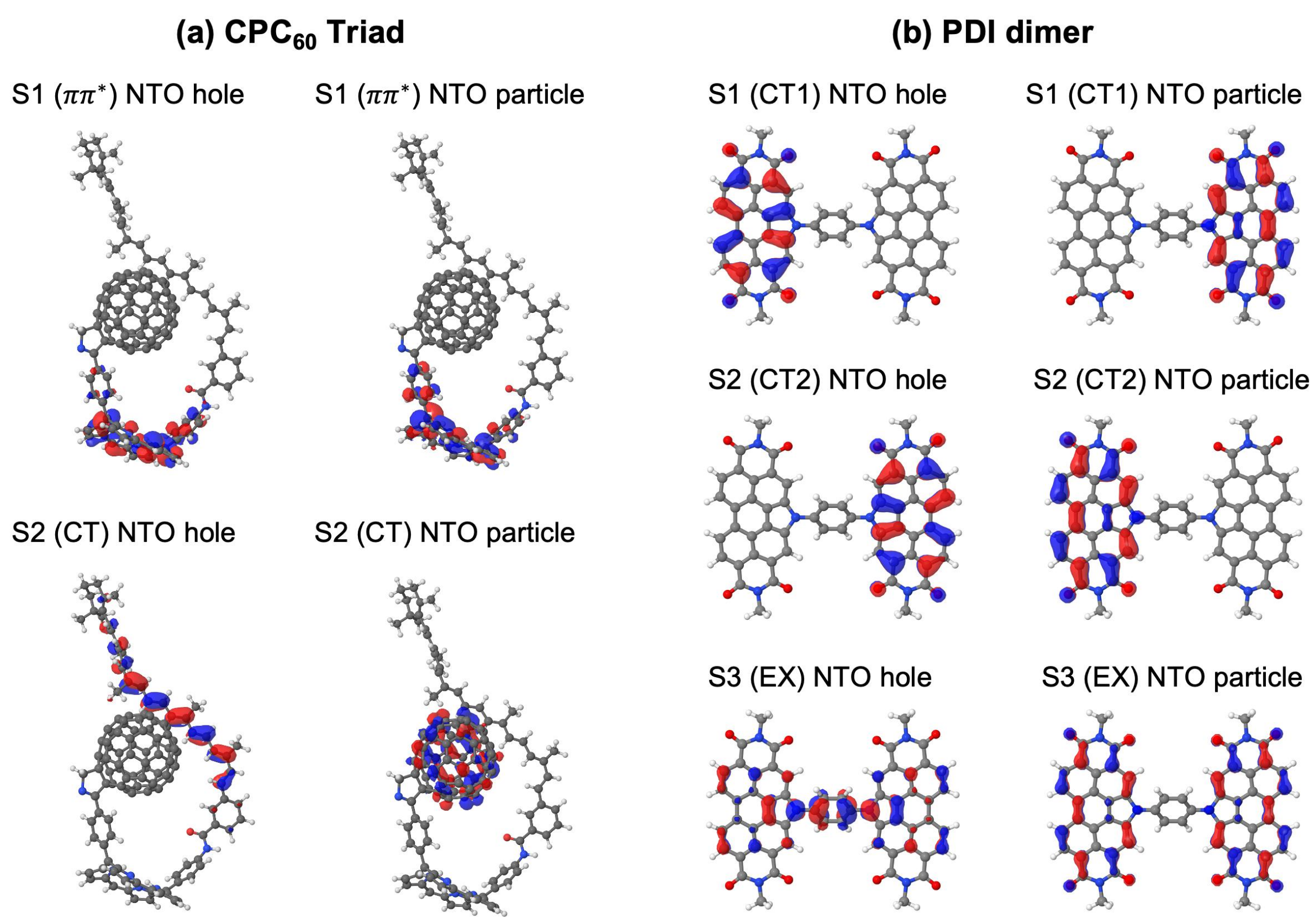

FIG. S1: Natural transition orbitals (NTOs) of (a) the $\pi\pi^*$ and CT states of the CPC_{60} triad and (b) the CT1, CT2, and EX states of the PDI dimer.

[a)]Electronic mail: ywzhang@nnu.edu.cn
[b)]Electronic mail: xiang.sun@nyu.edu

TABLE S1: Total charge (in e) of each moiety in the CPC_{60} triad on different electronic states, and the moieties include the carotenoid (C), the porphyrin (P), and the fullerene (C_{60}).

Model	State	Triad-C	Triad-P	Triad-C_{60}
AA	G	-0.20219957	0.42100846	-0.21880286
	$\pi\pi^*$	-0.19569148	0.42287349	-0.22718403
	CT	0.79194705	0.42064245	-1.21258445
UA	G	-0.20219958	0.42100845	-0.21880285
	$\pi\pi^*$	-0.19569147	0.42287347	-0.22718403
	CT	0.79194704	0.42064244	-1.21258445
2h	G	-0.20219957	0.42100845	-0.21880284
	$\pi\pi^*$	-0.19569146	0.42287348	-0.22718404
	CT	0.79194704	0.42064244	-1.21258445

TABLE S2: Total charge (in e) of each moiety in the PDI dimer on different electronic states, and the moieties include the left DPI (L), the bridge benzene (B), and the right PDI (R).

Model	State	PDI (L)	Bridge (B)	PDI (R)
AA	G	-0.11641107	0.22901205	-0.11259601
	EX	-0.21699885	0.43077364	-0.21377511
	CT1	0.88146671	0.20359531	-1.08506162
	CT2	-1.08795161	0.20316570	0.88478577
UA	G	-0.11641106	0.22901205	-0.11259600
	EX	-0.21699883	0.43077364	-0.21377510
	CT1	0.88146673	0.20359531	-1.08506164
	CT2	-1.08795156	0.20316569	0.88478577
2h	G	-0.11641106	0.22901206	-0.11259598
	EX	-0.21699886	0.43077363	-0.21377509
	CT1	0.88146672	0.20359530	-1.08506164
	CT2	-1.08795158	0.20316570	0.88478577

II. COMPUTATIONAL EFFICIENCY COMPARISON OF DIFFERENT COARSE-GRAINED LEVELS

This section outlines the numerical parameters and evaluates the computational efficiency gained by employing coarse-grained (CG) models for nonadiabatic molecular dynamics (NAMD) and classical molecular dynamics (MD) simulations.

A. Nonadiabatic molecular dynamics (NAMD) simulation parameters

The simulation details for the nonadiabatic dynamics tests of the triad in tetrahydrofuran (triad/THF) and the perylene diimide dimer in acetonitrile (PDI/ACN) systems are summarized in Table S3. The tested semiclassical mapping dynamics methods include mean-field (MF) Ehrenfest dynamics,[3] linearized semiclassical (LSC) mapping dynamics,[4–6] symmetrical quasiclassical (SQC) with triangle window[7,8] mapping dynamics, the extended classical mapping model (CMM),[9–11] and the spin-mapping model (SPM) with W-scheme.[12,13]

B. Wall Time of NAMD

To provide a consistent hardware comparison, all wall-clock times were measured using the GPU-accelerated QCDyn package[1] on a single NVIDIA RTX 3080 GPU. Table S4 reports the NAMD wall times across different structural resolutions evaluated using the MF method. For each system, 1250 independent trajectories were propagated for 15 ps, utilizing a nuclear time step of 0.001 ps (15000 steps total). This results in a cumulative simulated time of 18.75 ns per resolution. While coarse-graining reduces the total particle count and accelerates the simulations, the reduction in wall time scales sublinearly due to fixed algorithmic overheads.

TABLE S3: Numerical parameters for nonadiabatic dynamics simulation of the triad/THF and PDI/ACN systems on all CG levels, including the ZPE parameter γ, the nuclear time step Δt, the electronic time step δt, the number of trajectories N_{traj}, the length of each trajectory $t_{\max}$.

Method	ZPE parameter γ	Δt (fs)	δt (fs)	N_{traj}	$t_{\max}$ (ps)
		Triad/THF			
MF	0	1	0.05	1×10^4	15
LSC	1/2	1	0.05	5.0×10^4	15
SQC	1/3	1	0.05	3.25×10^4	15
CMM	1/2	1	0.05	1×10^4	15
SPM	$(\sqrt{F+1}-1)/F$	1	0.05	1×10^4	15
		PDI/ACN			
MF	0	1	0.05	5×10^4	5
LSC	1/2	1	0.02	5×10^4	5
SQC	1/3	1	0.02	5×10^4	5
CMM	1/2	1	0.05	1×10^4	5
SPM	$(\sqrt{F+1}-1)/F$	1	0.05	1×10^4	5

TABLE S4: NAMD wall time (in seconds) and particle number for different systems and CG resolutions.

System	Quantity	AA/AA	AA/UA	AA/3b	UA/UA	UA/3b	2h/UA
triad/THF	Wall time	52265	26443	20763	25421	21078	26409
	Particles	35307	13707	8307	13639	8239	13610
System	Quantity	AA/AA	AA/UA	AA/2b	UA/UA	UA/2b	2h/UA
PDI/ACN	Wall time	8017	5569	5482	5755	5475	5548
	Particles	8740	4420	2980	4392	2952	4366

C. Wall Time of MD

The explicit benchmark systems evaluated for NAMD were intentionally restricted to relatively small sizes to ensure that the fully atomistic AA/AA simulations remained computationally feasible as a reference. To better illustrate the true structural acceleration capacity of the CG models for macroscopic systems and to isolate this structural acceleration from the NAMD-specific coupled electronic–nuclear propagation, pure classical MD wall times were measured for large-scale solvent boxes. As detailed in Table S5, standard MD production runs of 50 ps with a 0.001 ps time step were performed for PDI/ACN systems containing one PDI dimer and 14,400 and 144,000 ACN molecules, respectively. Without the overhead of the nonadiabatic effects, the reduction in the classical MD wall time aligns much more closely with the theoretical reduction in particle number.

TABLE S5: MD wall time and particle number for PDI/ACN systems with different CG resolutions.

System	CG Level	Particles	Wall time
	AA	86500	39.03
14400 ACN	UA	43300	24.05
	2b	28900	22.13
	AA	864100	612.00
144000 ACN	UA	432100	308.80
	2b	288100	283.80

III. SUPPLEMENTAL FIGURES

In this section, we provide supplementary figures that further validate the nonadiabatic mapping dynamics and structural responses discussed in the main text.

Figure S2 presents the equilibrium and time-dependent RDFs of the PDI/ACN system in the ground (G), locally excited (EX), and charge transfer (CT1) states. These distributions are essential for completing the molecular picture of the transient solvent rearrangement observed in the main text. As shown in Figure S2(b,d), upon vertical excitation to the EX state, nearby ACN molecules rapidly reorient themselves such that their electronegative nitrogen atoms move significantly closer to the bridge

region relative to the ground state. Importantly, because the distribution of the ACN center of mass does not change significantly, this response is confirmed to be a localized reorientational motion rather than a bulk translational rearrangement of the solvent. Furthermore, the time evolution of the nonequilibrium RDF difference peak for the bridge-ACN(N) pair (Figure S2(e)) explicitly captures the two distinct timescales of this process: an initial rapid structural rise driven by the vertical excitation in the first 0.16 ps, followed by a slow decay as the electronic population subsequently transfers from the highly structured EX state to the CT2 state till 0.40 ps, after which the peak value of $\Delta g(r)$ stabilizes at around 0.19. This demonstrates that the initial structural dynamics are driven by a strong nonequilibrium transient response rather than static EX-to-CT2 charge differences.

Figures S3 and S4 compare the nonadiabatic population dynamics obtained from six different mapping methods (MF, LSC1, LSC2, SQC, CMM, and SPM) for the triad/THF and PDI/ACN systems, respectively. To evaluate method dependence, the fully atomistic AA/AA reference is compared against a selected robust coarse-grained model (UA/3b for the triad/THF system, and UA/2b for the PDI/ACN system). Across both physical systems, while the absolute decay rates vary depending on the chosen dynamical method, the qualitative population trends remain highly consistent between the atomistic and coarse-grained representations. For the triad system, the CG model faithfully reproduces the trend of the fully atomistic model in predicting the electronic transition from the $\pi\pi^*$ state to the CT state, with the minor exception that the SPM method exhibits a faster population decay rate in the CG model compared to the AA reference. For the PDI dimer, all methods accurately capture the asymmetric charge transfer that favors the CT2 state over CT1. The only noticeable difference is observed in the SQC dynamics, where the CG-level results show a slightly larger population decrease than the AA reference, with a deviation of approximately 5%. These results confirm that the ability of an appropriate CG model to reproduce atomistic physical trends is largely independent of the underlying semiclassical propagation scheme, notwithstanding minor method-specific deviations.

Figures S5 to S8 present the time-dependent explicit-molecule RDFs evaluated using LSC ($\gamma = 1/2$) and SQC ($\gamma = 1/3$) dynamics. These serve as supplementary tests to the MF-derived structural responses discussed in the main text. For the triad/THF system (Figs. S5 and S6), the LSC and SQC dynamics at the AA and UA levels reproduce the primary solvent structural responses observed in the MF dynamics, though the 2h representations exhibit severe noise due to structural inadequacy. Importantly, the triad system highlights the methodological differences between the LSC and SQC approaches. The origin of this difference lies in their initial sampling protocols. The LSC method uniformly samples the initial electronic mapping variables across all available electronic states. Consequently, the initial nuclear forces are highly averaged over multiple states rather than reflecting the potential energy surface (PES) of the predominantly populated locally excited state, rendering the early nuclear evolution less physical.[1] In contrast, the SQC method restricts its initial electronic mapping variable sampling strictly to the PES of the initially populated state. As a result, the initial nuclear forces generated by SQC are significantly more physically representative.

For the PDI/ACN system (Figs. S7 and S8), the LSC and SQC trajectories exhibit a similar structural contrast compared to the MF results, which show a slow solvent rearrangement after 1.0 ps. Ultimately, as previously noted in the literature,[1] while methods utilizing uniform mapping variable sampling (like LSC, CMM, and SPM) can maintain robust global electronic population dynamics, the resulting explicit nuclear dynamics and time-dependent RDFs become less physically descriptive than those generated via focused sampling or the MF Ehrenfest approach.

Figure S9 shows the NAMD for the PDI/ACN system with the original electronic couplings from the electronic structure calculation, which are obtained with the MSH models constructed from AA/AA and UA/2b explicit-molecule simulations. The symmetry-breaking charge transfer to CT2 state is seen on the 1 ns timescale, while CT1 is not populated on this timescale. Both MSH models show the identical population dynamics.

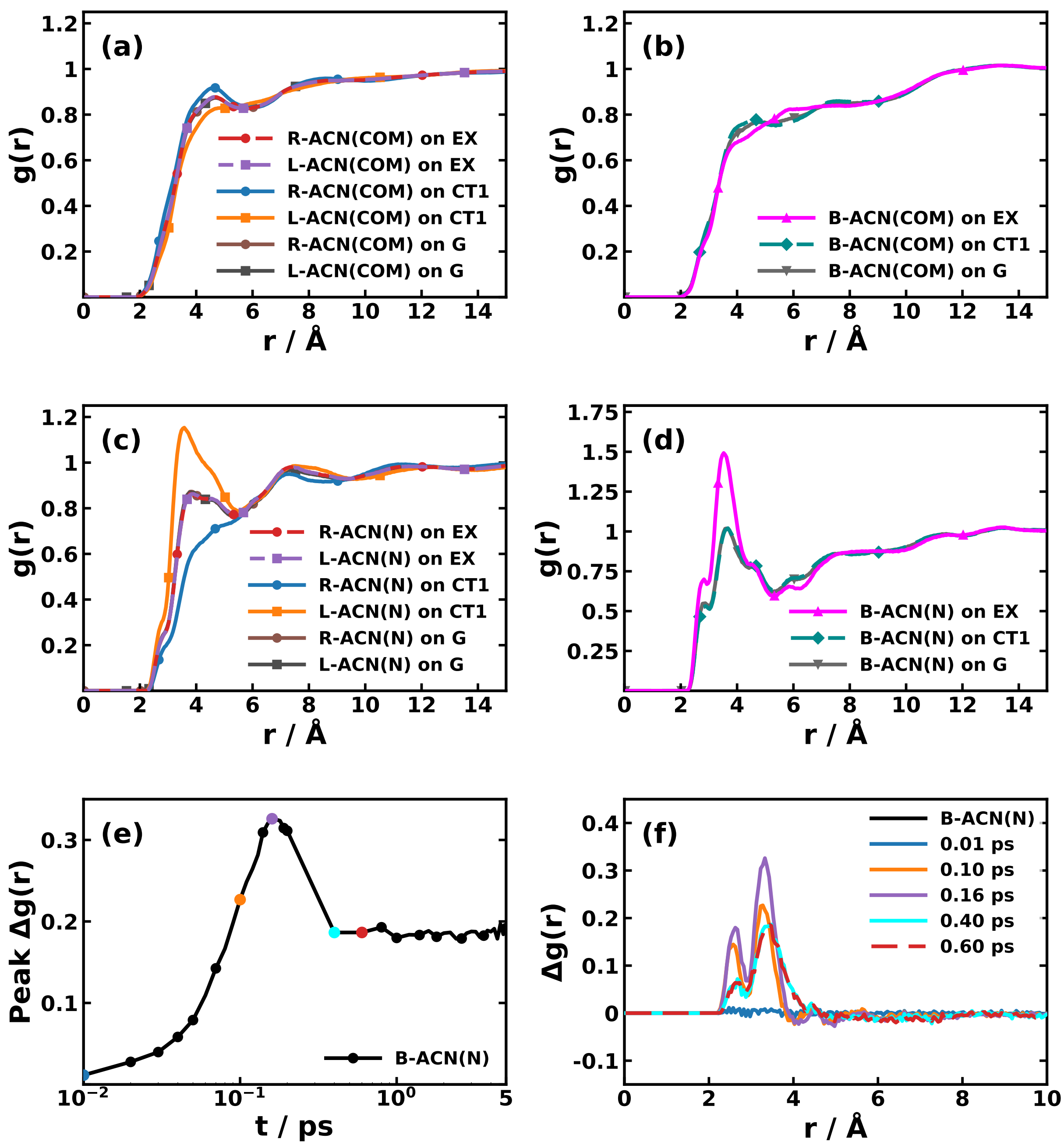

FIG. S2: Radial distribution functions (RDFs) between solute perylene diimide (PDI) dimer and solvent acetonitrile (ACN) in different electronic states, including ground (G), locally excited (EX), and charge transfer 1 (CT1) states. (a) Equilibrium RDFs between the left (L) and right (R) PDI moieties and the center of mass of ACN; (b) Equilibrium RDFs between the bridge benzene moiety (B) and the center of mass of ACN; (c) Equilibrium RDFs between the L and R moieties and the nitrogen atom of ACN; (d) Equilibrium RDFs between the bridge and the nitrogen atom of ACN; (e) Time evolution of the peak value of nonequilibrium $\Delta g(r)$ between the bridge and the nitrogen atom of ACN; (f) Time evolution of nonequilibrium $\Delta g(r)$ between the bridge and the nitrogen atom of ACN on a short timescale (0.6 ps).

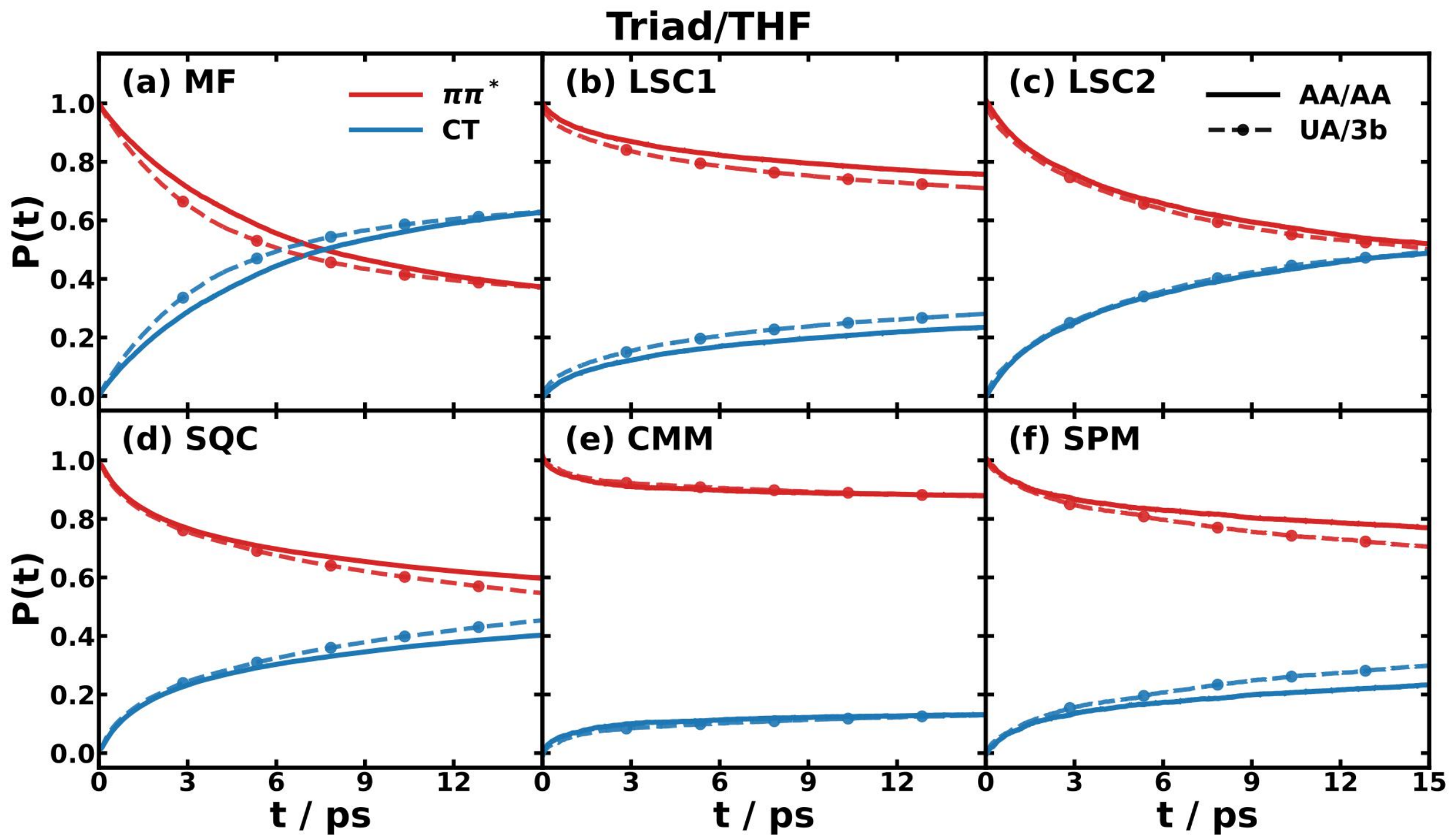

FIG. S3: Comparison of nonadiabatic dynamics of the triad/THF system represented on AA/AA (solid) and UA/3b (dashed) levels, simulated with different dynamical methods: (a) MF, (b) LSC1, (c) LSC2, (d) SQC with triangle window and $\gamma = 1/3$, (e) CMM with $\gamma = 1/2$, (f) SPM with W-scheme.

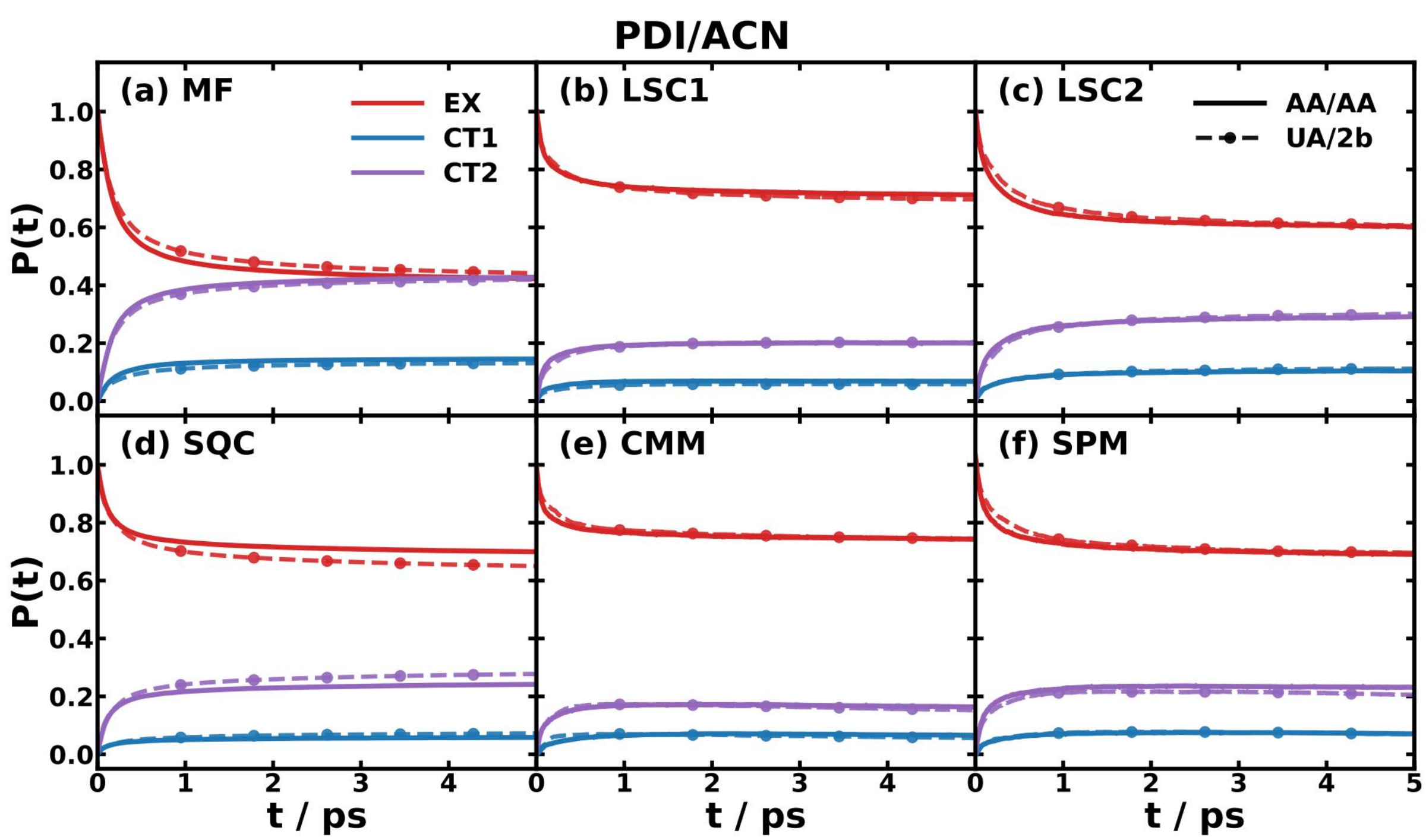

FIG. S4: Comparison of nonadiabatic dynamics of the PDI/ACN system represented on AA/AA (solid) and UA/2b (dashed) levels, simulated with different dynamical methods: (a) MF, (b) LSC1, (c) LSC2, (d) SQC with triangle window and $\gamma = 1/3$, (e) CMM with $\gamma = 1/2$, (f) SPM with W-scheme.

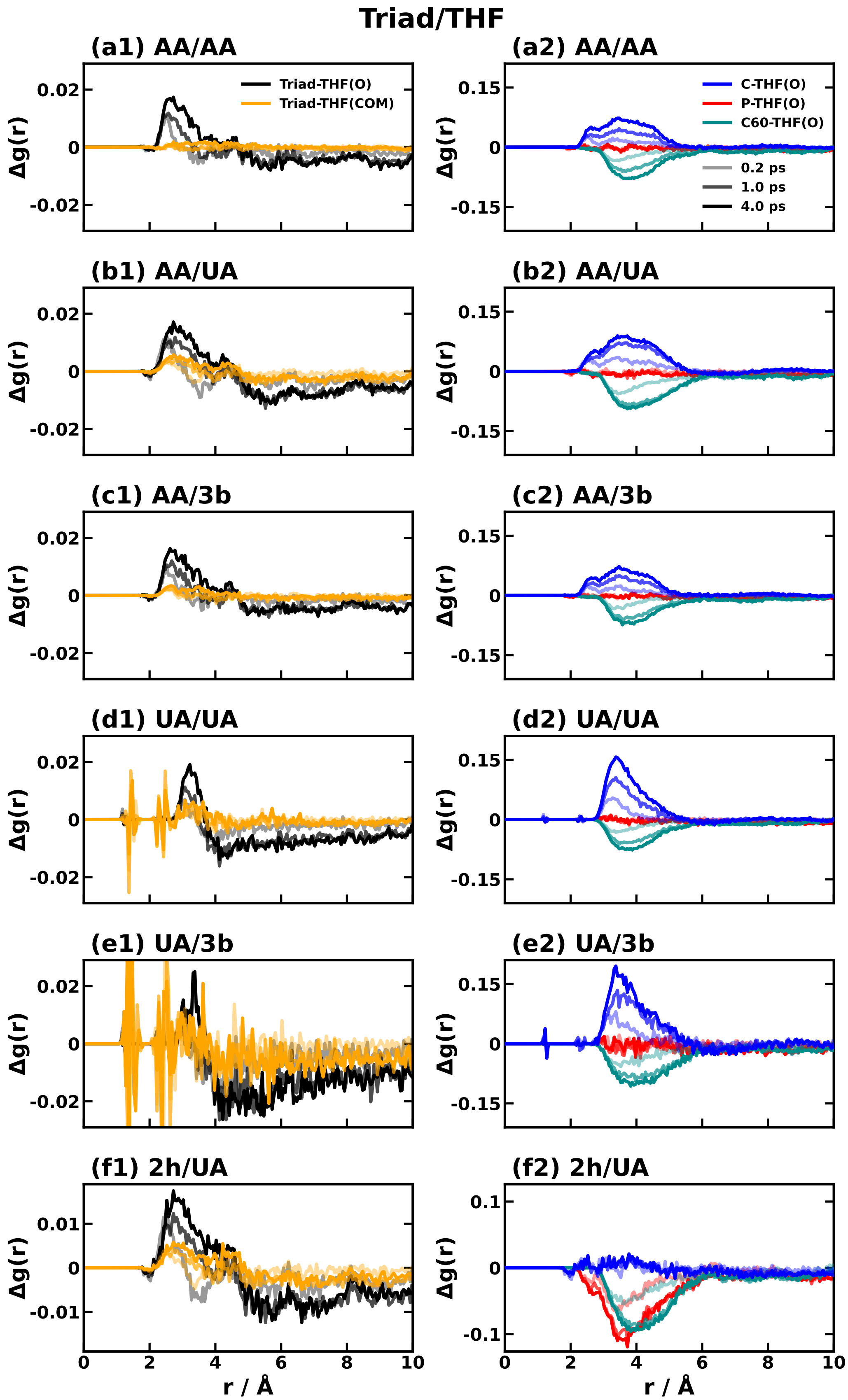

FIG. S5: Time-dependent radial distribution function (RDF) $\Delta g(r)$ of triad/THF system between times 0.2, 1.0, and 4.0 ps with respect to time zero after the photoexcitation, as obtained by LSC dynamics ($\gamma = 1/2$). Left panels show RDF evolution for the triad surface and the oxygen atom (O) of THF (black) and the triad surface and the center of mass (COM) of THF (orange); right panels show carotenoid (C, blue), porphyrin (P, red), and fullerene (C60, turquoise) moieties and oxygen of THF contributions.

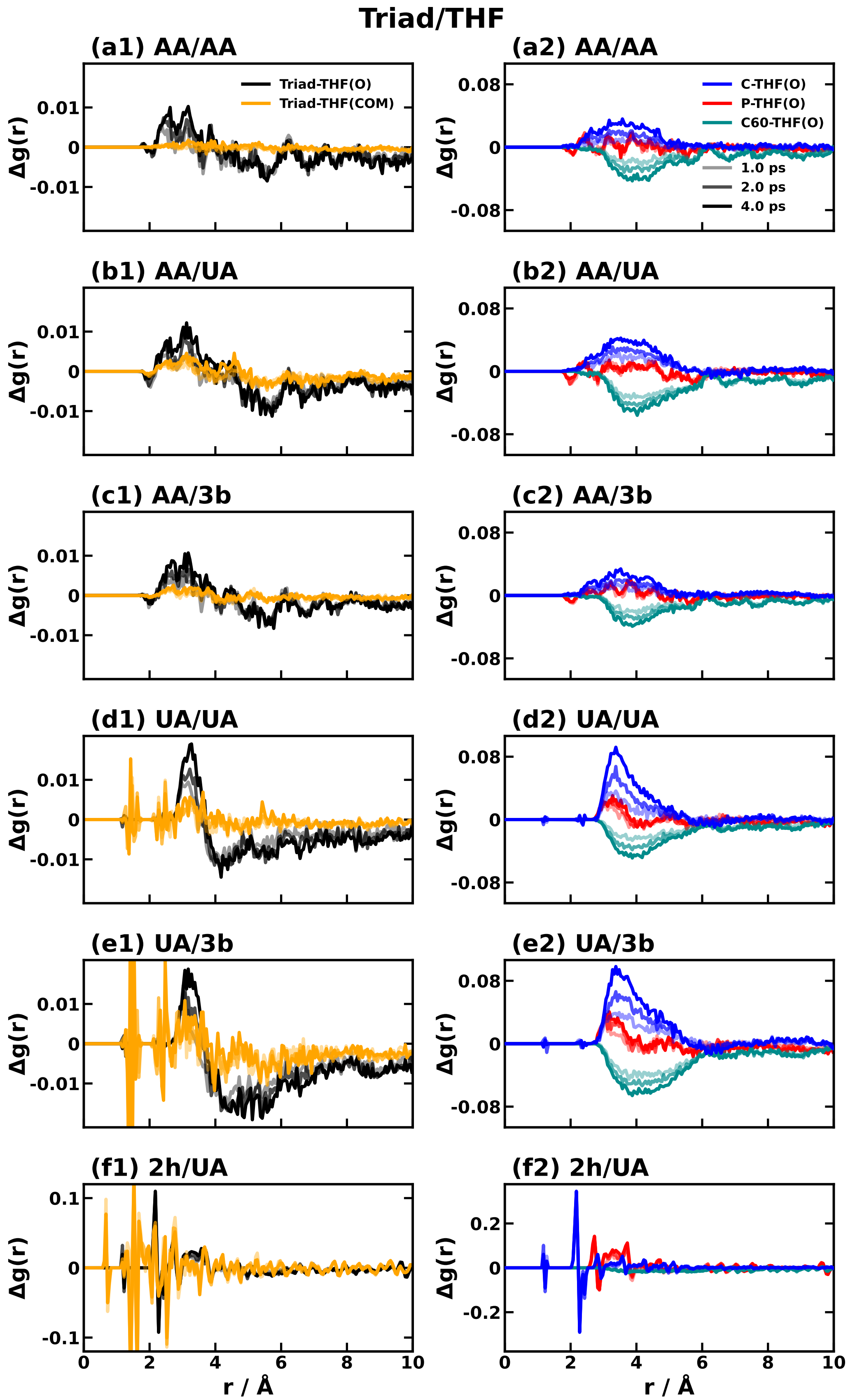

FIG. S6: Time-dependent radial distribution function (RDF) $\Delta g(r)$ of triad/THF system between times 1.0, 2.0, and 4.0 ps with respect to time zero after the photoexcitation, as obtained by SQC dynamics with triangle window ($\gamma = 1/3$). Left panels show RDF evolution for the triad surface and the oxygen atom (O) of THF (black) and the triad surface and center of mass (COM) of THF (orange); right panels show carotenoid (C, blue), porphyrin (P, red), and fullerene (C60, turquoise) moieties and oxygen of THF contributions.

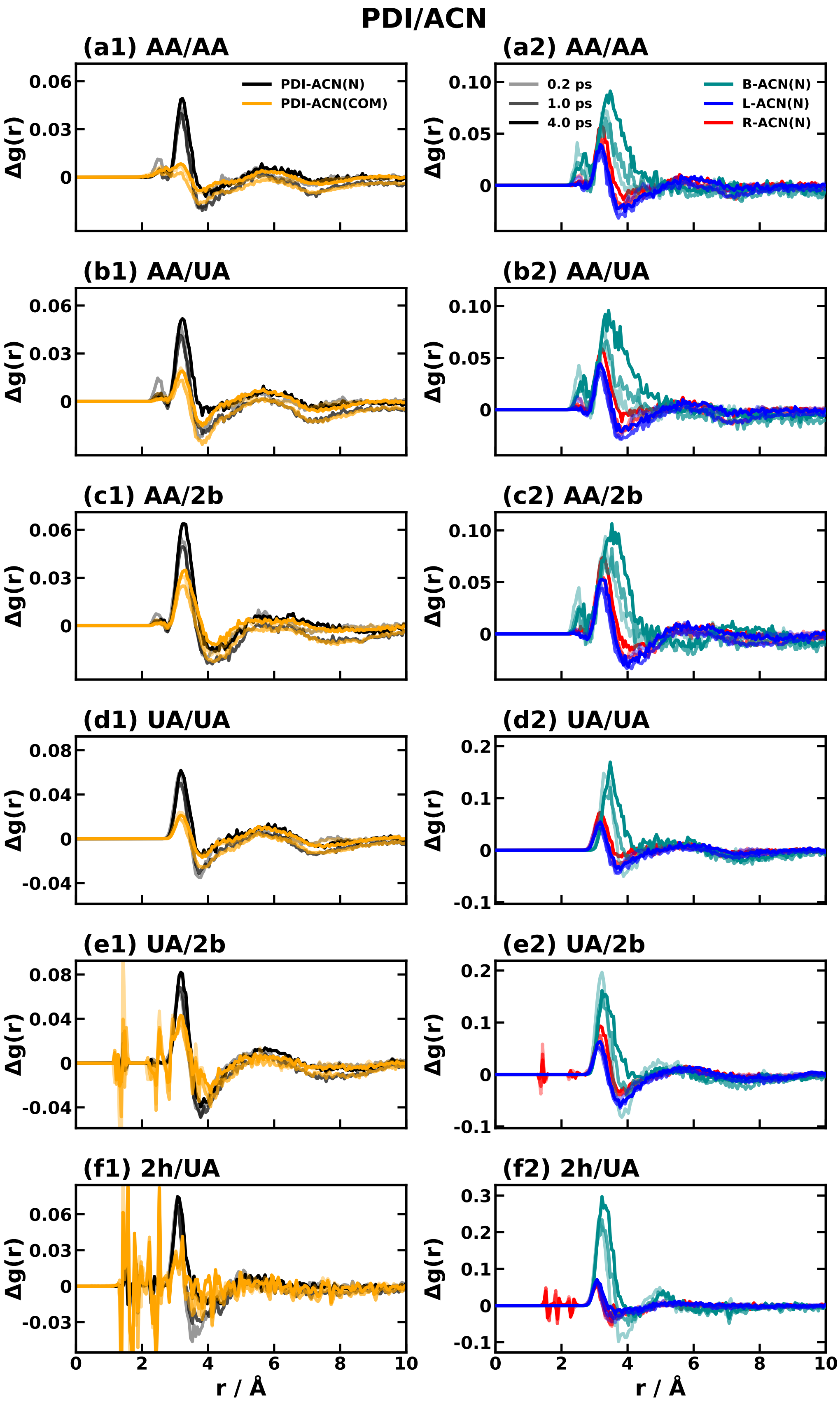

FIG. S7: Time-dependent radial distribution function (RDF) $\Delta g(r)$ of PDI/ACN system between times 0.2, 1.0, and 4.0 ps with respect to time zero after the photoexcitation, as obtained by LSC dynamics ($\gamma = 1/2$). Left panels show RDF evolution for PDI surface and the nitrogen atom (N) of ACN (black) and PDI surface and the center of mass (COM) of ACN (orange); right panels show the bridge (B, turquoise), left perylene diimide (L, blue), and right perylene diimide (R, red) moieties of PDI and nitrogen of ACN contributions.

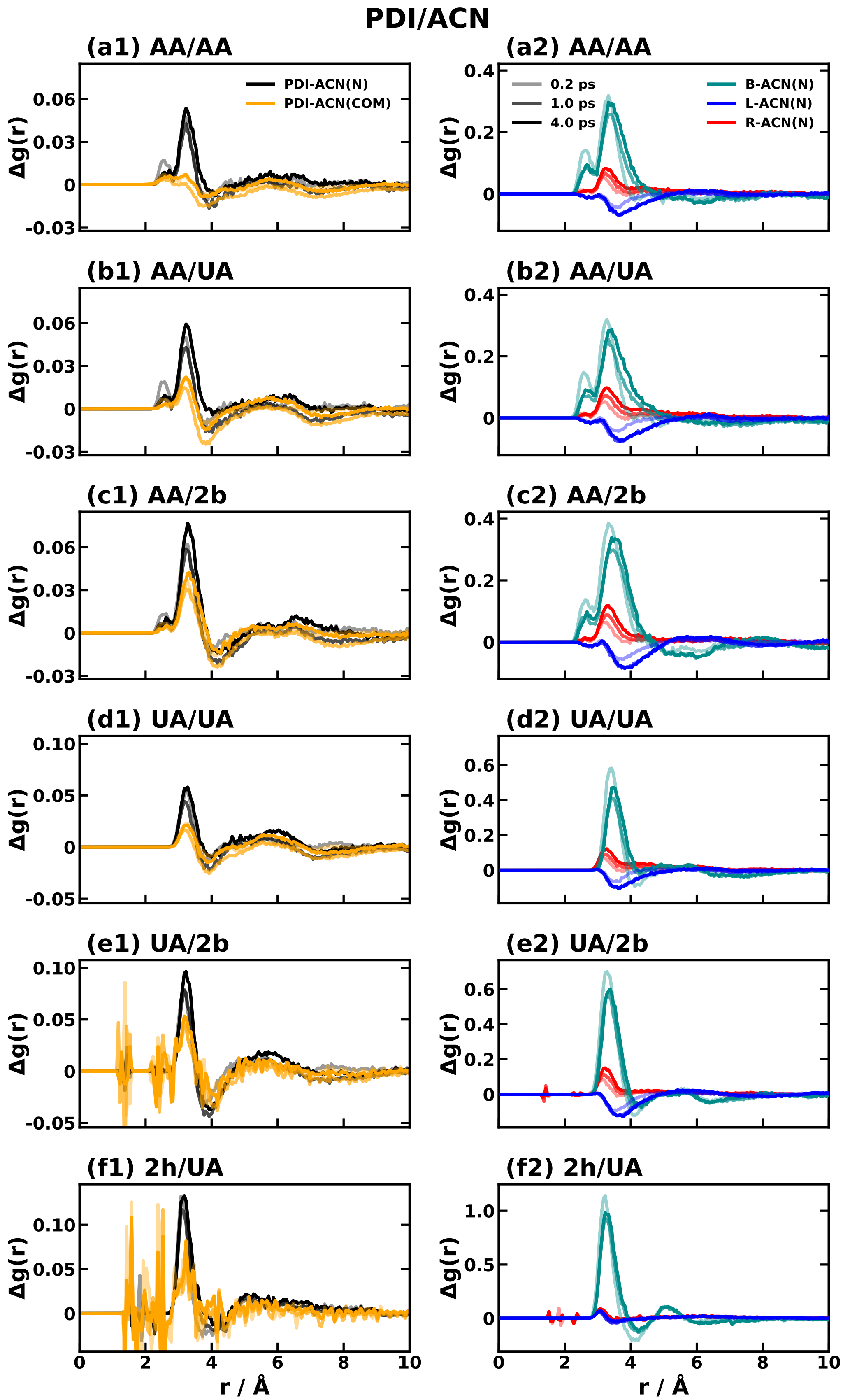

FIG. S8: Time-dependent radial distribution function (RDF) $\Delta g(r)$ of PDI/ACN system between times 0.2, 1.0, and 4.0 ps with respect to time zero after the photoexcitation, as obtained by SQC dynamics with triangle window ($\gamma = 1/3$). Left panels show RDF evolution for PDI surface and the nitrogen atom (N) of ACN (black) and PDI surface and the center of mass (COM) of ACN (orange); right panels show the bridge (B, turquoise), left perylene diimide (L, blue), and right perylene diimide (R, red) moieties of PDI and nitrogen of ACN contributions.

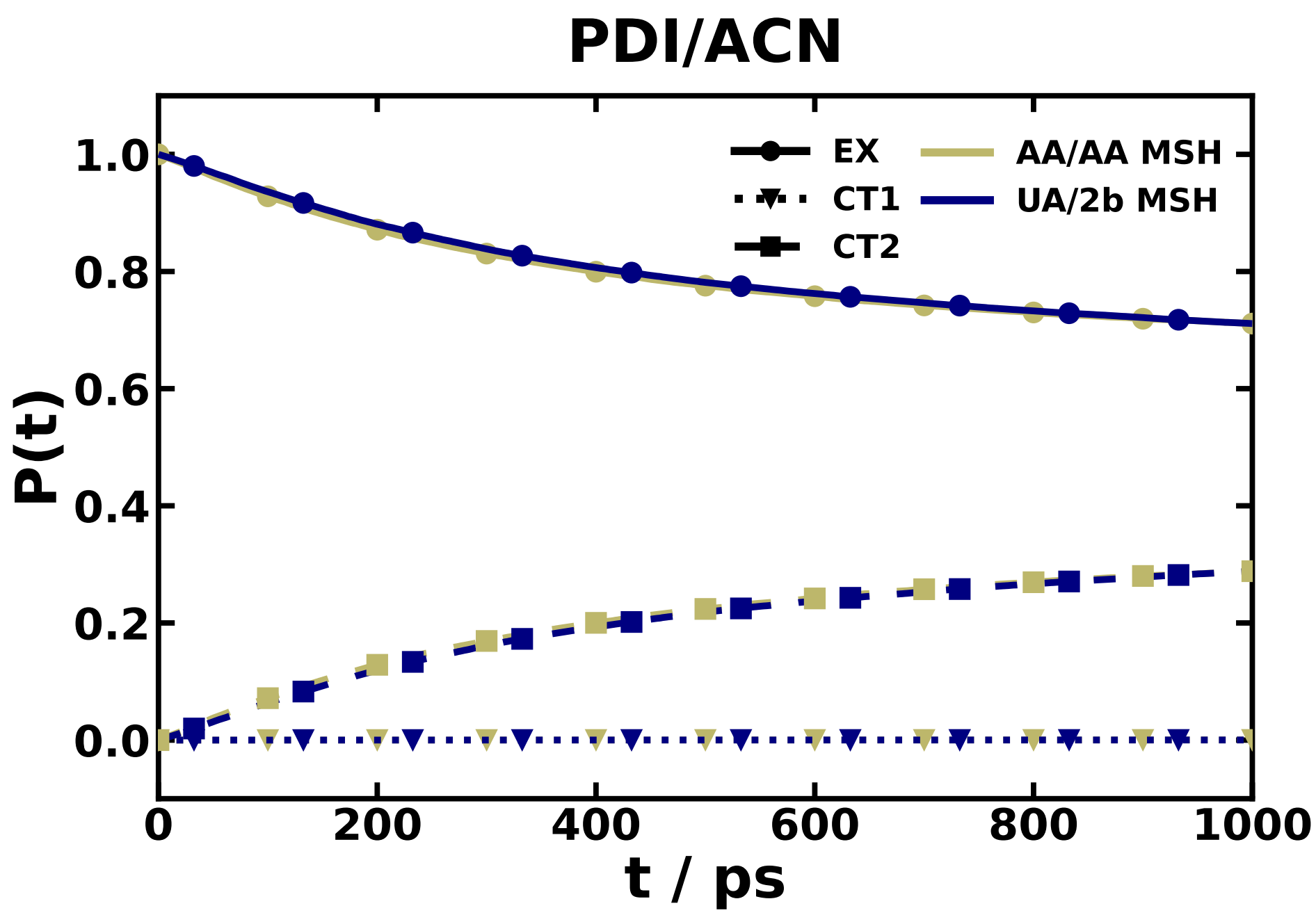

FIG. S9: Nonadiabatic dynamics of the PDI/ACN system using the MSH models constructed from AA/AA and UA/2b levels and using the original electronic coupling, $\Gamma_{\mathrm{EX,CT1}} = 9.3 \times 10^{-6}$ eV, $\Gamma_{\mathrm{EX,CT2}} = 2.8 \times 10^{-4}$ eV, and $\Gamma_{\mathrm{CT1,CT2}} = 3.3 \times 10^{-5}$ eV. NAMD were simulated with the MF dynamical method at 300 K and averaged over 1×10^{4} trajectories with a time step of 1 fs.

REFERENCES

[1] Z. Hu and X. Sun, "All-Atom Nonadiabatic Semiclassical Mapping Dynamics for Photoinduced Charge Transfer of Organic Photovoltaic Molecules in Explicit Solvents," J. Chem. Theory Comput. **18**, 5819–5836 (2022).

[2] Y. Guo, Z. Ma, X. Niu, W. Zhang, M. Tao, Q. Guo, Z. Wang, and A. Xia, "Bridge-Mediated Charge Separation in Isomeric N-Annulated Perylene Diimide Dimers," J. Am. Chem. Soc. **141**, 12789–12796 (2019).

[3] P. Ehrenfest, "Comment on the Approximate Validity of Classical Mechanics within Quantum Mechanics," Z. Phys. **45**, 455–457 (1927).

[4] X. Gao, M. A. C. Saller, Y. Liu, A. Kelly, J. O. Richardson, and E. Geva, "Benchmarking Quasiclassical Mapping Hamiltonian Methods for Simulating Electronically Nonadiabatic Molecular Dynamics." J. Chem. Theory Comput. **16**, 2883–2895 (2020).

[5] M. A. C. Saller, A. Kelly, and J. O. Richardson, "On the Identity of the Identity Operator in Nonadiabatic Linearized Semiclassical Dynamics," J. Chem. Phys. **150**, 071101 (2019).

[6] Z. Hu, D. Brian, and X. Sun, "Multi-State Harmonic Models with Globally Shared Bath for Nonadiabatic Dynamics in the Condensed Phase," J. Chem. Phys. **155**, 124105 (2021).

[7] S. J. Cotton and W. H. Miller, "Symmetrical Windowing for Quantum States in Quasi-Classical Trajectory Simulations: Application to Electronically Non-Adiabatic Processes," J. Chem. Phys. **139**, 234112 (2013).

[8] S. J. Cotton and W. H. Miller, "A New Symmetrical Quasi-Classical Model for Electronically Non-Adiabatic Processes: Application to the Case of Weak Non-Adiabatic Coupling," J. Chem. Phys. **145**, 144108 (2016).

[9] J. Liu, "A Unified Theoretical Framework for Mapping Models for the Multi-State Hamiltonian," J. Chem. Phys. **145**, 204105 (2016).

[10] X. He and J. Liu, "A New Perspective for Nonadiabatic Dynamics with Phase Space Mapping Models," J. Chem. Phys. **151**, 024105 (2019).

[11] X. He, Z. Gong, B. Wu, and J. Liu, "Negative Zero-Point-Energy Parameter in the Meyer-Miller Mapping Model for Nonadiabatic Dynamics," J. Phys. Chem. Lett. **12**, 2496–2501 (2021).

[12] J. E. Runeson and J. O. Richardson, "Spin-Mapping Approach for Nonadiabatic Molecular Dynamics," J. Chem. Phys. **151**, 044119 (2019).

[13] J. E. Runeson and J. O. Richardson, "Generalized Spin Mapping for Quantum-Classical Dynamics," J. Chem. Phys. **152**, 084110 (2020).
